# Evidence that regulation of intramembrane proteolysis is mediated by substrate gating during sporulation in *Bacillus subtilis*

**DOI:** 10.1371/journal.pgen.1007753

**Published:** 2018-11-07

**Authors:** Fernando H. Ramírez-Guadiana, Christopher D. A. Rodrigues, Kathleen A. Marquis, Nathalie Campo, Rocío del Carmen Barajas-Ornelas, Kelly Brock, Debora S. Marks, Andrew C. Kruse, David Z. Rudner

**Affiliations:** 1 Department of Microbiology and Immunobiology, Harvard Medical School, Boston MA United States of America; 2 Department of Systems Biology, Harvard Medical School, Boston, MA; 3 Department of Biological Chemistry and Molecular Pharmacology, Harvard Medical School, Boston, MA; Indiana University, UNITED STATES

## Abstract

During the morphological process of sporulation in *Bacillus subtilis* two adjacent daughter cells (called the mother cell and forespore) follow different programs of gene expression that are linked to each other by signal transduction pathways. At a late stage in development, a signaling pathway emanating from the forespore triggers the proteolytic activation of the mother cell transcription factor σ^K^. Cleavage of pro-σ^K^ to its mature and active form is catalyzed by the intramembrane cleaving metalloprotease SpoIVFB (B), a Site-2 Protease (S2P) family member. B is held inactive by two mother-cell membrane proteins SpoIVFA (A) and BofA. Activation of pro-σ^K^ processing requires a site-1 signaling protease SpoIVB (IVB) that is secreted from the forespore into the space between the two cells. IVB cleaves the extracellular domain of A but how this cleavage activates intramembrane proteolysis has remained unclear. Structural studies of the *Methanocaldococcus jannaschii* S2P homolog identified closed (substrate-occluded) and open (substrate-accessible) conformations of the protease, but the biological relevance of these conformations has not been established. Here, using co-immunoprecipitation and fluorescence microscopy, we show that stable association between the membrane-embedded protease and its substrate requires IVB signaling. We further show that the cytoplasmic cystathionine-β-synthase (CBS) domain of the B protease is not critical for this interaction or for pro-σ^K^ processing, suggesting the IVB-dependent interaction site is in the membrane protease domain. Finally, we provide evidence that the B protease domain adopts both open and closed conformations *in vivo*. Collectively, our data support a substrate-gating model in which IVB-dependent cleavage of A on one side of the membrane triggers a conformational change in the membrane-embedded protease from a closed to an open state allowing pro-σ^K^ access to the caged interior of the protease.

## Author summary

Regulated Intramembrane Proteolysis is a broadly conserved mechanism for transducing information across lipid bilayers. In these signaling pathways a protease on one side of the membrane triggers the activation of a membrane-embedded protease that cleaves its substrate within or adjacent to the cytoplasmic face of the membrane. Site-2 metalloproteases (S2P) are the most commonly used intramembrane cleaving proteases in these pathways but the mechanism by which cleavage on one side of the membrane triggers intramembrane proteolysis remains poorly understood. Here, we provide evidence for a substrate-gating model in which an extracellular signaling protease triggers a conformational change in a S2P family member from a closed to an open conformation allowing its substrate access to the catalytic center of the enzyme.

## Introduction

Regulated Intramembrane Proteolysis (RIP) is a broadly used strategy to transduce information across lipid bilayers [1–3]. In many of these RIP pathways, proteolysis on one side of the membrane by a so-called site-1 protease leads to the activation of a membrane-embedded site-2 protease that cleaves and activates a substrate on the other side of the membrane, ultimately leading to the activation of gene expression. Although the signaling (site-1) and intramembrane cleaving (site-2) proteases have been identified in many of these pathways, how intramembrane proteolysis is regulated at the molecular level remains poorly understood.

The zinc metalloproteases of the Site-2 Protease (S2P) family are among the most commonly used intramembrane cleaving proteases in RIP signaling pathways [4–6]. This family is composed of four subfamilies that share a conserved catalytic core [7]. Well-characterized members include the mammalian Site-2 Protease (S2P) [8] and *E*. *coli* RseP [9, 10], both from subfamily 1, and the *B*. *subtilis* sporulation protease SpoIVFB (B) [11–13] from subfamily 3. There are currently no characterized members of subfamilies 2 and 4. Mammalian S2P is involved in signaling pathways that sense and respond to intracellular levels of sterols and misfolded proteins in the endoplasmic reticulum [5]. In both cases, the canonical two-step protease cascade results in intramembrane proteolysis of membrane-anchored transcription factors and their release into the cytoplasm, translocation into the nucleus, and activation of gene expression. *E*. *coli* RseP and orthologs in diverse bacterial phyla are involved in envelope stress response pathways [4, 6]. These pathways commonly involve extracytoplasmic function (ECF) sigma factors that are held inactive by membrane-anchored anti-sigma factors. Specific envelope stresses trigger site-1 cleavage of the anti-sigma factor on the extracytoplasmic side of the membrane followed by RseP-mediated intramembrane proteolysis and activation of the ECF sigma factor. *B*. *subtilis* SpoIVFB (referred to as B, for simplicity) is involved in cell-cell signaling during the morphological process of sporulation [14] and is the subject of this study.

**Fig 1:**
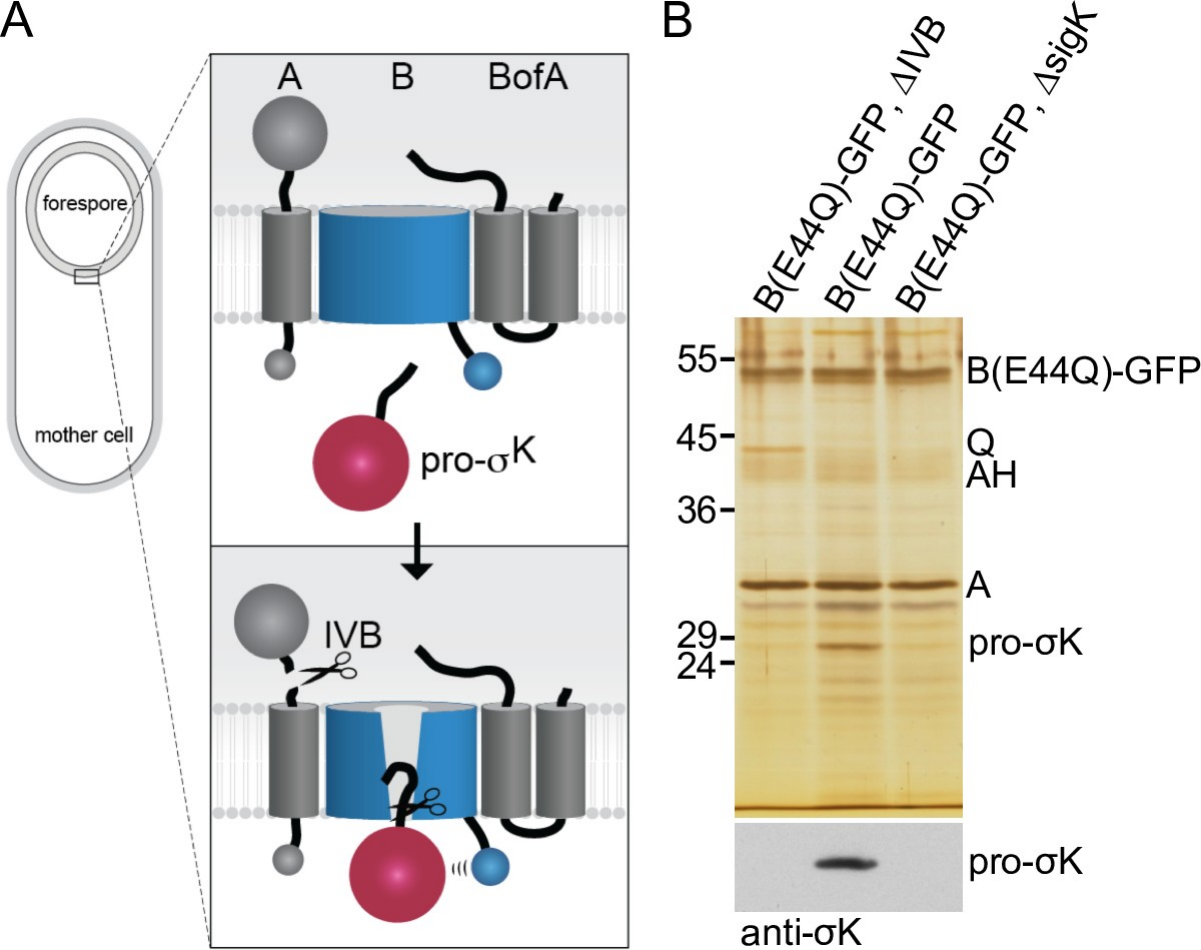
Pro-σ^K^ resides in a membrane complex with the B protease in a IVB-dependent manner. **(A)** Schematic model of regulated intramembrane proteolysis during sporulation. Prior to forespore signaling, A and BofA hold B in a closed conformation in the membranes surrounding the forespore. IVB-dependent cleavage of A triggers a conformational change in B allowing pro-σ^K^ access to the catalytic center of the protease. Pro-σ^K^ processing then leads to late mother cell gene expression and spore maturation (not depicted in the diagram). For simplicity, A and BofA are shown interacting with B but not each other. Co-immunoprecipitation and cytological assays [33] suggest A and BofA interact in the absence of B. **(B)** Silver-stained gel of immunoprecipitated proteins from detergent-solubilized membrane preparations of the indicated strains at hour 4 of sporulation. Proteins indicated on the right were identified by mass spec or from immunoprecipitations using mutant strains. Immunoblot of the same samples using anti-σ^K^ antibodies is shown below.

In response to starvation, *B*. *subtilis* differentiates into a dormant spore [15–17]. The first morphological event in this process is the formation of a polar septum that divides the sporulating cell into a large mother cell and smaller forespore. Shortly after division, the mother cell membranes migrate around the forespore generating a cell within a cell (**Fig 1A**). The mother then packages the forespore in protective layers while the spore prepares for dormancy. Upon spore maturation, mother cell lysis releases the stress-resistant spore into the environment. Throughout this morphological process, the mother cell and forespore follow distinct programs of developmental gene expression that are linked to each by cell-cell signaling pathways [18, 19]. The final communication between the forespore and mother cell involves a RIP signaling pathway in which an inactive mother cell transcription factor (pro-σ^K^) is proteolytically activated by the S2P protease family member B in response to a signal from the forespore [11, 12, 14]. The B protease is produced in the mother cell at an earlier stage in sporulation and localizes to the mother-cell membranes that surround the forespore [11, 12, 20]. B is held inactive at this site by two membrane proteins SpoIVFA (A) and BofA [14, 21] (**Fig 1A**). Upon the completion of engulfment, a site-1 signaling protease called SpoIVB (IVB) is produced in the forespore and secreted into the space between the two membranes that separate mother cell and forespore where it cleaves the extracytoplasmic domain of A [22–25] (**Fig 1A**). This cleavage relieves inhibition imposed on B, triggering pro-σ^K^ processing and the activation of late mother cell gene expression. Thus, in this RIP pathway, the site-1 signaling protease (IVB) cleaves a negative regulator of the S2P family protease (B). How A and BofA hold B inactive and how cleavage by IVB triggers relief of inhibition remain unclear.

The mechanisms by which site-1 cleavage activates intramembrane proteolysis by S2P family members are largely unknown. The pathway for which regulation is most well understood is the one that employs *E*. *coli* RseP. Site-1 cleavage of the periplasmic domain of the anti-sigma factor RseA triggers RseP-mediated intramembrane proteolysis of RseA and activation of the ECF sigma factor σ^E^ [4]. RseP and S2P subfamily 1 members contain extracytoplasmic PDZ domains [7, 26]. *In vitro* studies have found that the interaction between this domain and the exposed C-terminal residue of RseA generated by site-1 cleavage is critical for intramembrane proteolysis [27]. However, *in vivo* analysis indicates that this is likely to be only a part of the story [28]. *In vivo*, the PDZ domain on RseP appears to function as a size-exclusion filter to prevent substrates with large extracytoplasmic domains from gaining access to the active site of the membrane protease. In addition, a membrane-reentrant β-loop in RseP has been shown to participate in the recognition of the substrate transmembrane helix and its destabilization for presentation to the protease active site [29]. The absence of a high-resolution structure of a member of this subfamily has prevented a complete mechanistic picture of this regulatory pathway.

The only published S2P structures come from *Methanocaldococcus jannaschii* (mjS2P) [30], a member of subfamily 3. Unlike S2P and RseP, the proteases in this subfamily lack extracytoplasmic PDZ domains. Subfamily 3 members contain six N-terminal transmembrane helices that make up the membrane protease domain followed by a C-terminal cystathionine-β-synthase (CBS) domain [7, 31, 32]. The mjS2P structures comprised the N-terminal protease domain that shares 29% identity (54% similarity) with the membrane protease domain of the *B*. *subtilis* B protease. Two molecules of mjS2P were present in the asymmetric unit of the crystal forming an antiparallel pseudo-dimer. One of the monomers was in a "closed" conformation in which the active site was surrounded by transmembrane helices and thus inaccessible to substrate. The other protomer adopted a relatively open conformation, in which the first and sixth transmembrane segments were laterally displaced by 10–12 Å, generating a deep groove that runs the length of the molecule. Based on these findings, it was hypothesized that mjS2P could transition between closed and open states to allow substrates access to the active site of the protease, however the biological relevance of these conformations has never been established. Here, using the *B*. *subtilis* sporulation RIP pathway, we investigate this substrate-gating model. Our analysis provides evidence that A and BofA hold the membrane protease B in a closed conformation and IVB cleavage triggers a shift to the open conformation allowing pro-σ^K^ access to the interior of the protease.

## Results

### Stable association between B and pro-σ^K^ requires IVB

In previous studies we used co-immunoprecipitation to investigate the interaction between B and its two negative regulators A and BofA [22, 33]. To prevent activation of the membrane-embedded protease, these experiments were performed in strains lacking the site-1 signaling protease IVB. Although we could efficiently recover the processing complex, we never detected pro-σ^K^ in the immunoprecipitates. Prompted by the two structural conformations of the mjS2P protease domain [30], we wondered whether A and BofA hold B in a closed conformation preventing an interaction with pro-σ^K^ and potentially IVB signaling could generate an open conformation allowing stable association between protease and substrate. To investigate this possibility, we repeated the co-immunoprecipitations comparing strains with and without IVB. To prevent pro-σ^K^ processing and release of the mature transcription factor, the B protease contained the catalytic mutant E44Q [11, 12]. In addition, B(E44Q) was fused to GFP, which we previously found stabilizes the protease from degradation upon IVB signaling [33]. The strains were induced to sporulate and harvested at hour 4 of sporulation. A crude membrane preparation was solubilized with the non-ionic detergent digitonin and B(E44Q)-GFP was immunoprecipitated with anti-GFP antibody resin. The immunoprecipitated material was then analyzed by SDS-PAGE and silver staining.

As reported previously [22, 33], we efficiently immunoprecipitated B-GFP and its regulator A (**Fig 1B**). BofA is a small (9 kDa) protein and is likely present in the dye front (**S1A Fig**). In support of the idea that the processing complex is anchored in the mother cell membranes that surround the forespore by SpoIIIAH (AH) and SpoIIQ (Q) [34], these proteins were also present in the immunoprecipitates (**Fig 1B** and **S1 Fig**). Furthermore, consistent with published work showing that the Q protein is a substrate of the IVB signaling protease [22, 35], the level of co-precipitated Q was lower from the *IVB*^*+*^ strain. For unknown reasons, the levels of co-precipitated A were similar in the presence and absence of IVB, despite being a substrate of the signaling protease. Importantly, a protein band similar in size to pro-σ^K^ (27 kDa) was present in the immunoprecipitate from the *IVB*^+^ strain but absent from the Δ*IVB* mutant (**Fig 1B**). This protein was also absent in an immunoprecipitate from a *IVB*^+^ strain that lacked *sigK* (**Fig 1B**). To determine whether this protein was indeed pro-σ^K^, we excised the band from the silver-stained gel and the corresponding region from the Δ*IVB* and Δ*sigK* lanes, digested the proteins with trypsin, and analyzed the products by Mass Spectrometry. Three unique peptides (see Methods) corresponding to pro-σ^K^ were identified in the immunoprecipitate from the *IVB*^+^ strain while none was detected from the Δ*IVB* and Δ*sigK* immunoprecipitates. Finally, immunoblot analysis using anti-σ^K^ antibodies identified pro-σ^K^ specifically in the immunoprecipitate from the *IVB*^+^ strain (**Fig 1B**). Collectively, these data suggest that stable association between pro-σ^K^ and the B-A-BofA processing complex requires the IVB signaling protease.

### Pro-σ^K^-CFP localization to the outer forespore membrane requires IVB protease activity

**Fig 2:**
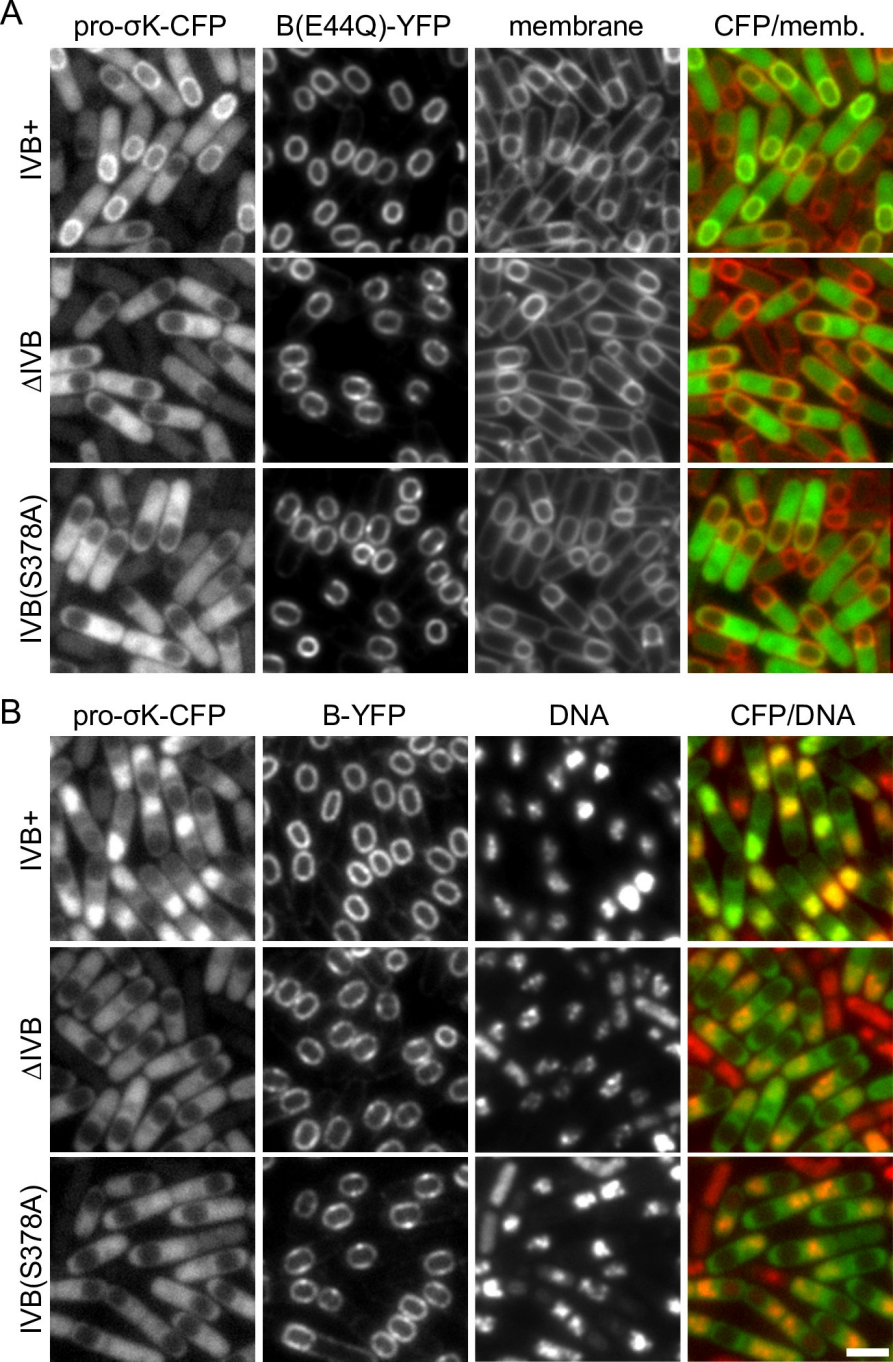
Pro-σ^K^-CFP localization to the membranes surrounding the forespore requires IVB protease activity. Representative images of the indicated strains at hour 4 of sporulation. **(A)** Pro-σ^K^-CFP co-localizes with B(E44Q)-YFP in the mother-cell membranes surrounding the forespore in cells harboring the IVB signaling protease. In the absence of IVB or a catalytic mutant (S378A) of IVB, pro-σ^K^-CFP localizes to the mother-cell cytoplasm with some enrichment on the nucleoid. Membranes were stained with the fluorescent dye TMA-DPH. Larger fields of cells over a sporulation time course and quantification of forespore-associated pro-σ^K^-CFP can be found in **S3** and **S4 Figs**. **(B)** Pro-σ^K^-CFP co-localizes with the mother-cell nucleoid in strains harboring the catalytically active B (site-2) protease and the IVB (site-1) signaling protease. In the absence of IVB or in the catalytic mutant IVB(S378A), pro-σ^K^ localizes in the mother-cell cytoplasm with some enrichment on the nucleoid. DNA was stained with DAPI. Images were scaled identically. Scale bar indicate 2 μm.

To investigate whether a similar phenomenon could be observed *in vivo*, we used fluorescence microscopy, taking advantage of a functional fusion between pro-σ^K^ and the cyan fluorescent protein (CFP) (**S2E Fig**). To simultaneously visualize the B membrane protease while also protecting it from degradation, we fused B(E44Q) to YFP. Importantly, a fusion of the wild-type B protease to YFP supported efficient sporulation (**S2E Fig**) and processing of both pro-σ^K^ and pro-σ^K^-CFP (**S2A** and **S2B Fig**). Strains harboring these fusions were induced to sporulate and then monitored by fluorescence microscopy over a sporulation time course (**Fig 2** and **S3 Fig**). Previous immunofluorescence microscopy and cell fractionation studies indicate that pro-σ^K^ non-specifically associates with membranes *in vivo* [36]. However, in sporulating cells lacking the IVB signaling protease, pro-σ^K^-CFP appeared to localize in the mother cell cytoplasm with weak enrichment on the nucleoid, presumably due to non-specific interactions with the chromosome. We suspect pro-σ^K^-CFP transiently associates with the lipid bilayer but this interaction is too short-lived to be detected in live cells. By contrast and as expected, in *IVB*^+^ cells harboring a wild-type B-YFP fusion, mature σ^K^-CFP co-localized with the mother-cell nucleoid (**Fig 2B)** consistent with its proteolytic processing (**S2B Fig**) and association with core RNA polymerase [36]. Importantly, in >80% of the sporulating cells harboring wild-type IVB and the B(E44Q) catalytic mutant, pro-σ^K^-CFP accumulated in the mother cell membranes surrounding the forespore (**Fig 2, S3** and **S4 Figs**). This localization pattern provides further evidence that stable association between pro-σ^K^ and its processing complex depends upon IVB. Finally, pro-σ^K^-CFP failed to localize in the outer forespore membrane in a strain harboring a catalytic mutant (S378A) of IVB [25] (**Fig 2** and **S4B Fig**) indicating that IVB protease activity is required for the stable interaction between pro-σ^K^ and the signaling complex.

### Pro-σ^K^-CFP localization to the outer forespore membrane requires B but not A or BofA

**Fig 3:**
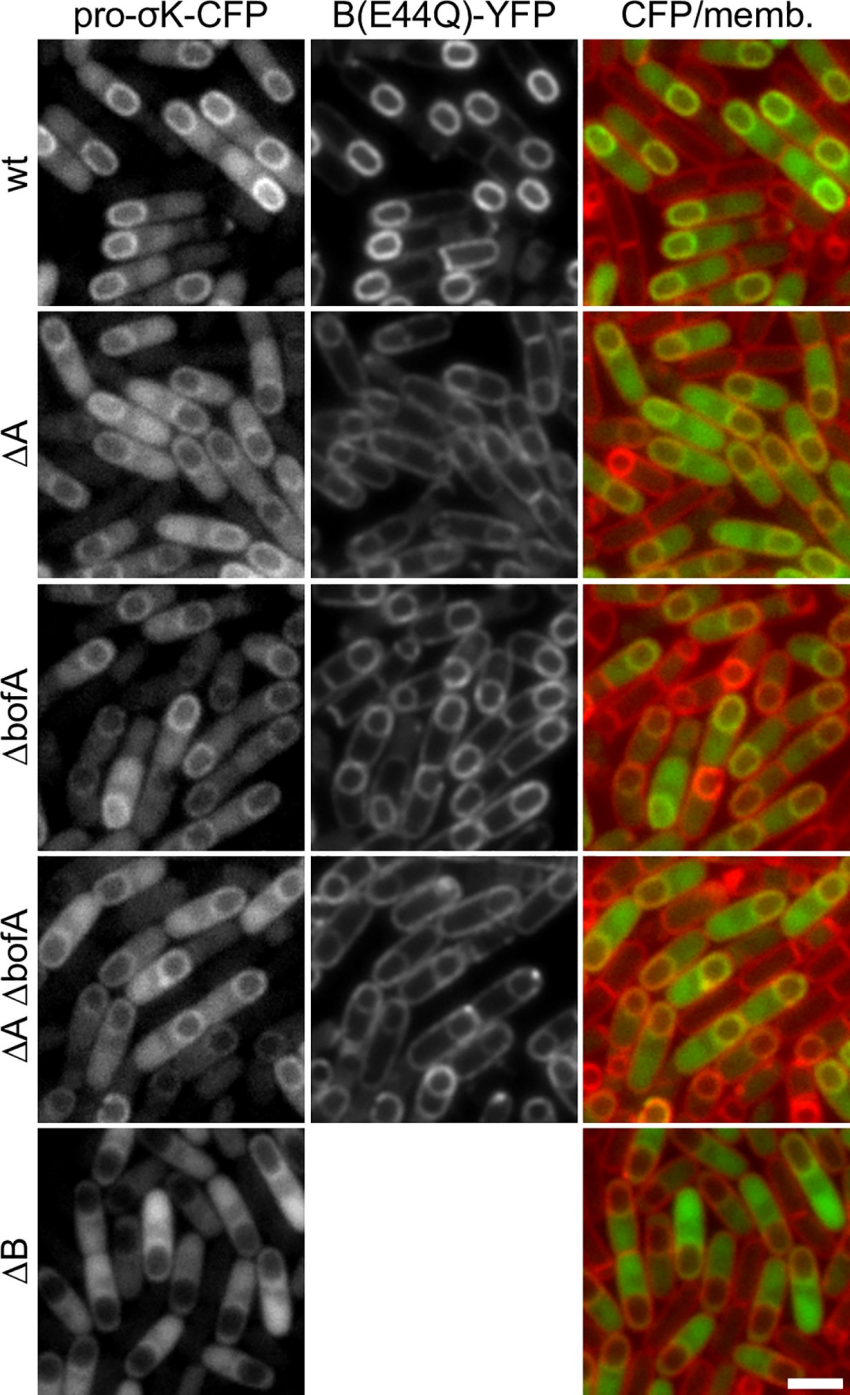
Pro-σ^K^-CFP localization to the membranes surrounding the forespore requires the B protease. Representative images of the indicated strains at hour 4 of sporulation. Pro-σ^K^-CFP localizes to the membranes surrounding the forespore in cells harboring B(E44Q)-YFP, in the absence of A, BofA, or both. In sporulating cells lacking the B protease, pro-σ^K^ localizes to the mother-cell cytoplasm with some enrichment on the nucleoid. The cytoplasmic pro-σ^K^-CFP signal is more pixelated in the strains lacking A and/or BofA compared to the Δ*B* mutant (see text). Images were scaled identically. Scale bar indicates 2 μm. Quantification of forespore-associated pro-σ^K^-CFP and larger fields of cells for all 5 strains over a sporulation time course can be found in **S4** and **S5 Figs**.

B, A, and BofA reside in a multimeric membrane complex in the outer forespore membrane [33] (**Fig 1A**). To investigate which of these factors tethers pro-σ^K^ to the complex, we analyzed pro-σ^K^-CFP localization in sporulating cells lacking each of these components. In all cases, these strains possessed an intact IVB signaling protease and, other than the Δ*B* mutant, the B(E44Q)-YFP fusion. As previously reported [33, 37], in sporulating cells lacking A, BofA, or both, the B protease was no longer exclusively anchored in the outer forespore membrane and instead was distributed in all mother cell-derived membranes (**Fig 3** and **S5 Fig**). In these mutants, pro-σ^K^-CFP was detectable in the membranes surrounding the forespore (**Fig 3** and **S5 Fig**). The pro-σ^K^-CFP signal was reduced compared to wild-type presumably due to the lower levels of B(E44Q) present in the forespore membrane. However, the percentage of sporulating cells with forespore-associated pro-σ^K^-CFP was similar to wild-type (**S4C Fig**). Surprisingly, pro-σ^K^-CFP did not appear to localize in the peripheral membranes of the mother cell. The results of experiments described in the next section suggest that some of the pro-σ^K^-CFP fusion protein is present in these membranes but the signal is too weak to generate a membrane signal. Finally, in cells lacking the B protease, pro-σ^K^-CFP localization phenocopied the IVB null and was largely present in the cytoplasm with some enrichment on the nucleoid (**Fig 3, S4C** and **S5 Figs**). Taken together with the data presented in Figs 1 and 2, these experiments suggest that pro-σ^K^ specifically associates with the membrane-embedded protease and does so in a IVB-dependent manner.

### Membrane localization of pro-σ^K^ in vegetatively growing cells requires B and is lost when A and BofA are co-expressed

**Fig 4:**
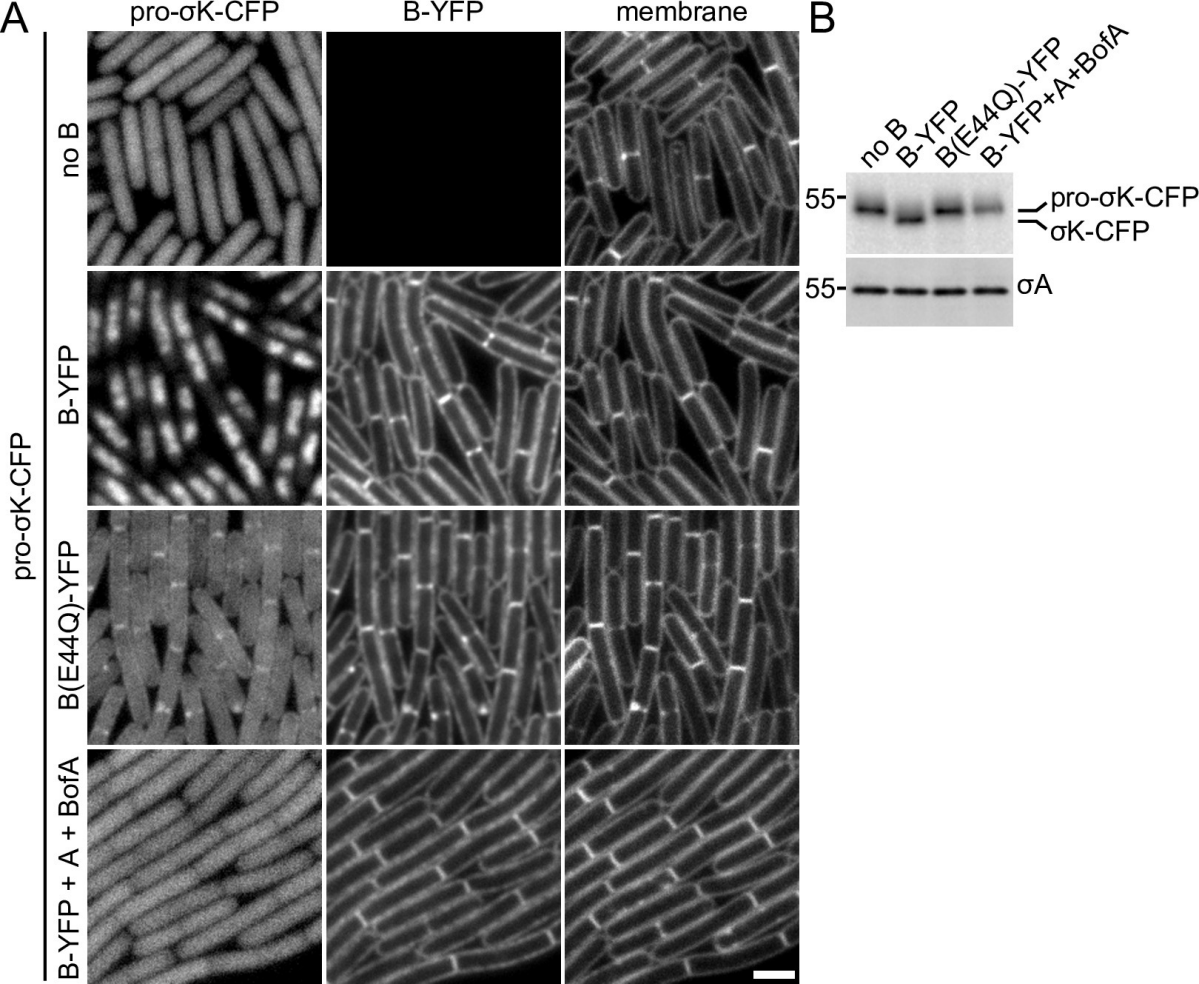
Pro-σ^K^-CFP localizes to the membranes of vegetatively growing cells when B(E44Q) is co-expressed. **(A)** Representative images of the indicated strains after 1.5 hours of induction. In the absence of the B protease, pro-σ^K^-CFP localizes in the cytoplasm. In the presence of wild-type B-YFP, pro-σ^K^-CFP gets processed and localizes to the nucleoid. Pro-σ^K^-CFP co-localizes with B(E44Q)-YFP in the septal and cytoplasmic membranes when co-expressed during vegetative growth. Pro-σ^K^-CFP remains cytoplasmic when wild-type B-YFP is co-expressed with its inhibitors A and BofA. The cytoplasmic Pro-σ^K^-CFP signal is weaker and more pixelated in the strain expressing B(E44Q)-YFP compared to those lacking B or co-expressing A and BofA (see text). All images were scaled identically. Larger fields of cells over the induction time course can be found in **S6 Fig.** Scale bar indicates 2 μm. **(B)** Immunoblot of the same strains in (A) using anti-σ^K^ antibodies showing pro-σ^K^-CFP processing in cells expressing B-YFP but not in cells expressing the E44Q mutant, the inhibitors A and BofA, or in a strain that does not express B during vegetative growth. σ^A^ was used to control for loading. Molecular weight markers (in kDa) are indicated to the left. An anti-GFP immunoblot to assess the relative amount of liberated (free) CFP in the four strains can be found in **S2C Fig**.

To determine whether additional sporulation-specific proteins were required for the association between B and pro-σ^K^, we engineered strains to express pro-σ^K^-CFP and B(E44Q)-YFP under IPTG control during vegetative growth. Exponentially growing cells were analyzed by fluorescence microscopy in a time-course after the addition of IPTG (**Fig 4** and **S6 Fig**). In cells lacking the B protease, pro-σ^K^-CFP appeared cytoplasmic (**Fig 4A** and **S6 Fig**). Co-expression of pro-σ^K^-CFP and wild-type B-YFP in the absence of its negative regulators A and BofA resulted in the production of processed σ^K^-CFP (**Fig 4B**) that co-localized with the nucleoid (**Fig 4A** and **S6 Fig**). In cells expressing the B(E44Q) catalytic mutant, pro-σ^K^-CFP appeared cytoplasmic with some localization at division septa and along the cytoplasmic membranes. Although the membrane signal was weak, we note that the pro-σ^K^-CFP signal in cells that were in contact with each other along their length had no gap in fluorescence between them as compared to the gaps observed for cytoplasmic pro-σ^K^-CFP in cells lacking the B protease (**Fig 4A**). We further note that the pro-σ^K^-CFP signal that appeared to be cytoplasmic in the B(E44Q) mutant was weaker and more pixelated than the signal observed in the cells lacking B, even though the levels of pro-σ^K^-CFP were similar in the two strains (**Fig 4B**). Both phenomena are consistent with the association of pro-σ^K^-CFP with B(E44Q) at the cell membrane. Importantly, pro-σ^K^-CFP remained largely full-length with relatively little free CFP liberated from the fusion (**S2C Fig**). The cytoplasmic pro-σ^K^-CFP signal was similarly weaker and more pixelated in sporulating cells lacking A, BofA, or both compared to the Δ*B* mutant in Fig 3. We suspect that this reduction in signal similarly reflects an association between pro-σ^K^-CFP and B(E44Q) in the peripheral mother-cell membranes despite our inability to directly detect the membrane signal.

Finally, we investigated whether expression of A and BofA in vegetatively-growing cells producing wild-type B-YFP is sufficient to inhibit pro-σ^K^-CFP processing and membrane association of the fluorescent fusion. As anticipated, pro-σ^K^-CFP remained full-length (**Fig 4B** and **S2C Fig**) and failed to associate with the membrane (**Fig 4A**). Collectively, these experiments support the model that A and BofA maintain B in a conformation that cannot interact with pro-σ^K^ and IVB-dependent signaling promotes a stable association between B and its substrate.

### IVB-dependent localization of pro-σ^K^-CFP in the absence of the CBS domain

The B protease, like other members the S2P group 3 subfamily, contains a C-terminal cytoplasmic cystathionine-β-synthase (CBS) domain [7]. These domains commonly bind ligands with adenosyl groups like ATP, AMP, S-adenosylmethionine, and c-di-AMP and regulate enzymatic or transport functions [31, 32]. *In vitro*, the CBS domain from the B protease has been shown to bind pro-σ^K^ [13]. We therefore wondered whether the stable association between B and pro-σ^K^ in response to IVB signaling was mediated by the CBS domain. Consistent with this idea, a deletion of this domain was reported to abolish pro-σ^K^ processing in an *E*. *coli* expression system [13, 38].

**Fig 5:**
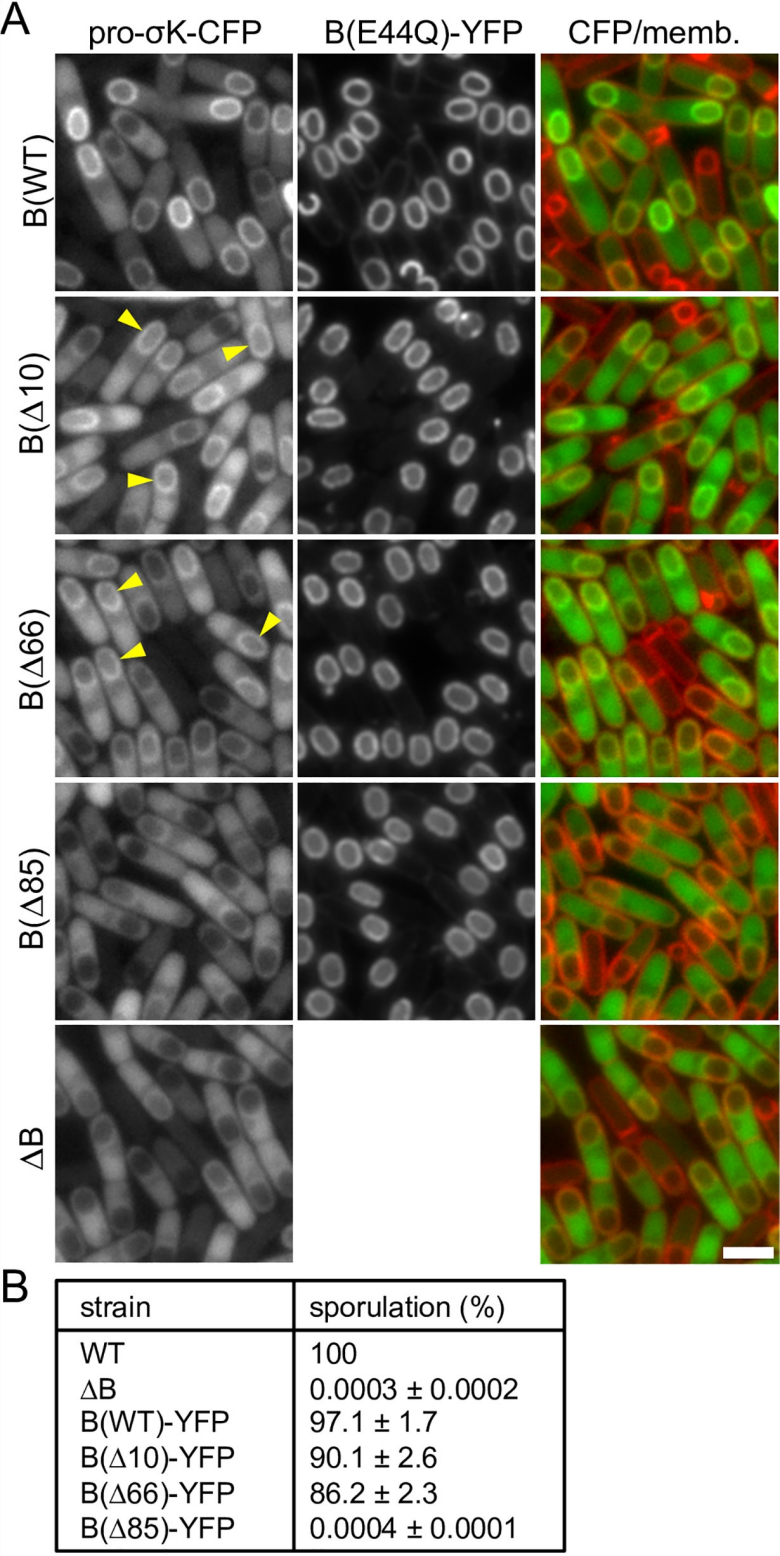
The CBS domain of the B protease is not critical for pro-σ^K^-CFP localization or efficient sporulation. **(A)** Representative images of the indicated strains at hour 4.5 of sporulation. Pro-σ^K^-CFP localizes around the forespore in cells producing B(E44Q)-YFP or 10 and 66 amino acid C-terminal truncations of the catalytic mutant. Pro-σ^K^-CFP localizes to the mother-cell cytoplasm with some enrichment on the nucleoid in the presence of a larger 85 amino acid truncation similar to sporulating cells lacking the B protease. Yellow carets highlight the localization of pro-σ^K^-CFP around the forespore. All images were scaled identically. Quantification of forespore-associated pro-σ^K^-CFP and larger fields of cells for all 5 strains over a sporulation time course can be found in **S4** and **S7 Figs**. Scale bar indicates 2 μm. **(B)** Table of sporulation efficiencies of the indicated strains (*n* = 3). All strains have untagged pro-σ^K^ and the B protease fusions have intact catalytic residues.

To investigate the role of the CBS domain in IVB-dependent signaling in *B*. *subtilis*, we generated a deletion (BΔ85) that lacks the CBS domain and interdomain linker and a smaller 66 amino acid deletion (BΔ66) that lacks the CBS domain but retains the linker [13, 38]. In addition, we generated a 10 amino acid C-terminal deletion (BΔ10) that was previously shown to be produced in *E*. *coli* at levels similar to wild-type but was impaired in pro-σ^K^ processing in this heterologous system [13]. All three deletions contained the E44Q catalytic mutation and were fused to YFP to monitor localization and help stabilize the proteins. All three fusion proteins localized properly (**Fig 5A**) and, based on fluorescence intensities, were produced at levels similar to the full-length protein (**S7B Fig**). Importantly, in sporulating cells harboring either the BΔ66 or BΔ10 mutant, pro-σ^K^-CFP accumulated in the outer forespore membranes (**Fig 5A** and **S7A Fig**), consistent with a recent study in which B truncations similar to those used here were found to interact with pro-σ^K^ when co-expressed in *E*. *coli* [38]. In support of the idea that the CBS domain helps stabilize the interaction between B and pro-σ^K^ [13, 38], the pro-σ^K^-CFP fluorescent signal around the forespore was reduced compared to wild-type, however and importantly the percentage of cells with forespore-localized pro-σ^K^-CFP was similar (**S4D Fig**). Moreover, we found that this localization pattern was dependent on the IVB signaling protein (**S8 Fig**). By contrast and similar to previous work [13], the BΔ85 mutant phenocopied the Δ*B* null. In line with our pro-σ^K^-CFP localization data, the BΔ10 and BΔ66 truncations with an intact protease domain supported efficient sporulation while the BΔ85 mutant sporulated at levels similar to the null (**Fig 5B**). Analysis of σ^K^ activity during a sporulation time course (**S9A Fig**) revealed that the BΔ66 and BΔ10 strains activate σ^K^ with kinetics similar to wild-type B. Furthermore, σ^K^ activity was dependent on IVB, indicating that these truncations are subject to inhibition by A and BofA and are activated by site-1 signaling. Finally, immunoblot analysis revealed that the BΔ66 mutant processed pro-σ^K^ to mature σ^K^ with slightly reduced efficiency compared to the matched wild-type control (**S9B Fig**). Altogether, these data indicate that the CBS domain is not critical for the IVB-dependent forespore localization of pro-σ^K^, pro-σ^K^ processing, or efficient spore formation. Thus, these results suggest that pro-σ^K^ associates with the membrane protease domain of B and raised the possibility that IVB triggers a transition from a closed to open conformation of the caged protease allowing stable association between pro-σ^K^ and B's catalytic center (**Fig 1A**).

### Evidence that B adopts open and closed conformations *in vivo*

**Fig 6:**
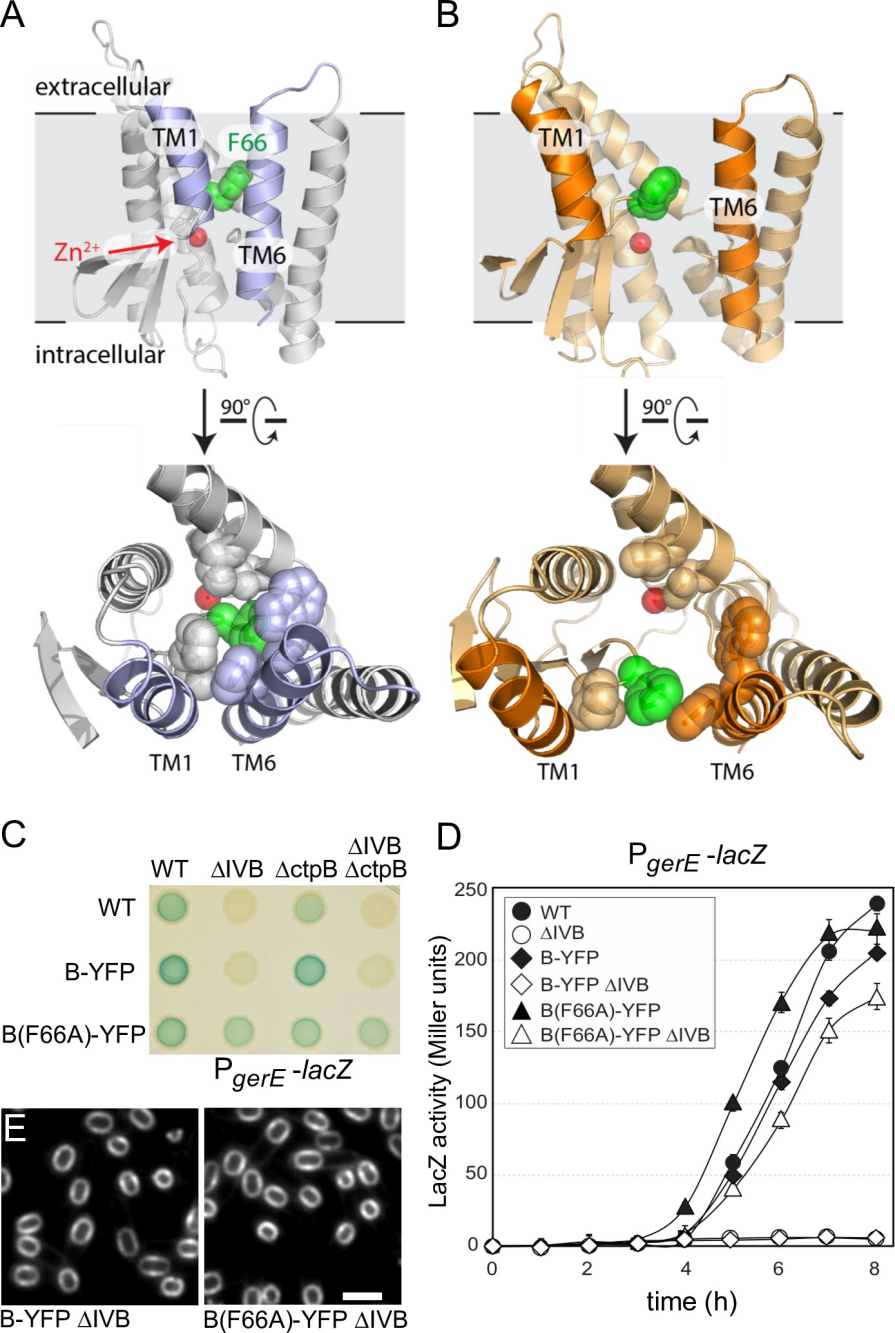
Evidence that B adopts open and closed conformations *in vivo*. Models of the closed **(A)** and open **(B)** conformations of the B protease domain based on the mjS2P structures. Phenylalanine 66 (green) in the membrane-reentrant β-loop that is predicted to occlude substrate and help stabilize the closed conformation and the catalytic Zn^2+^ ion (red ball) are indicated. Open and closed conformations rotated 90˚ are shown below. The residues in the catalytic core that are within 5 Å of F66 in the closed conformation are shown in both states. **(C)** B(F66A)-YFP activates σ^K^ in the absence of IVB and an auxiliary signaling protease CtpB. σ^K^ activity was monitored using a σ^K^-responsive promoter (P_*gerE*_) fused to *lacZ*. Image of a sporulation agar plate containing X-gal with the indicated strains after 24 hours of incubation at 37˚C. **(D)** Liquid β-galactosidase assay during a sporulation time course (*n* = 3). Only B(F66A) activates σ^K^ in the absence of IVB (open triangles). Liquid β-galactosidase assays of additional strains and immunoblot analysis of pro-σ^K^ processing can be found in **S11 Fig**. **(E)** B(F66A)-YFP localizes to the membranes surrounding the forespore. Representative images of the indicated strains at hour 4.5 of sporulation. Scale bar indicates 2 μm. Additional controls can be found in **S12 Fig**.

Prompted by these data, we sought to explore the biological relevance of the two conformations observed in the mjS2P structures. We used homology modeling to predict the structure of the B protease domain in both open and closed states (**Figs 6A** and **6B**). Because the B protease domain shares relatively low sequence identity with mjS2P, we used evolutionary co-variation analysis to guide target-template alignment and validate the resulting models. Evolutionary co-variation analysis takes advantage of the fact that residues that interact with one another in a folded protein tend to co-evolve to maintain their interactions [39, 40]. Analysis of mjS2P by this method using 5,290 S2P subfamily 3 orthologs identified extensive co-variation in amino acids that interact in the crystal structures (**S10 Fig**). Importantly, the interactions between the two molecules of mjS2P that form the antiparallel pseudo-dimer in the asymmetric unit of the crystal were not observed by co-variation analysis (**S10 Fig**), consistent with the anti-parallel interface being a crystallographic artifact. Co-variation analysis of B identified 81 evolutionary coupled residues (90% probability threshold) (**S10 Fig**). The analysis confirmed our assignment of sequence register in each of the TM segments and indicates that our homology models are reasonable proxies for the B structures.

Examination of the models of the B protease showed that a hydrophobic membrane-reentrant β-hairpin is buried in the hydrophobic core of the protease in the closed state (**Fig 6A**). Phenylalanine 66 sits at the tip of this β-hairpin, where it makes extensive interactions with a cluster of hydrophobic residues in the closed conformation while being mostly exposed in the open-conformation model (**Fig 6A** and **6B**). Specifically, F66 contacts V189, F188, W185, L136, and V128 in the closed state and only an interaction with F188 is preserved in the open state model. Interactions between F66 and these residues are predicted to occlude substrate and help stabilize the closed state of the lateral gate (**Fig 6A**). The analogous residue (I77) in the mjS2P protein is similarly buried in the core of the protein and is predicted to help stabilize the closed conformation. In the open conformation, F66 (and I77 in mjS2P) is displaced from the catalytic center and the first and sixth transmembrane segments have moved apart. To investigate whether the B protease domain adopts the closed conformation *in vivo*, we generated a B(F66A) mutant. Removal of the phenyl ring, which makes most of the hydrophobic contacts, is predicted to destabilize the closed state relative to the open, generating a constitutively active protease. In support of this idea, a strain harboring B(F66A)-YFP was capable of activating σ^K^ during sporulation in the absence of both the site-1 signaling protease IVB and an auxiliary signaling protease CtpB [22, 41] (**Fig 6C** and **6D** and **S11A Fig**). The B(F66A) point mutant similarly supported pro-σ^K^ processing in the absence of IVB albeit with reduced efficiency compared to a wild-type IVB^+^ strain (**S11B Fig**). In the presence of IVB, the point mutant had a modest sporulation defect (**S11C Fig**), consistent with the pre-mature activation of σ^K^ observed in this background (**Fig 6D**). Importantly, B(F66A)-YFP specifically localized in the membranes surrounding the forespore (**Fig 6E**) in a manner that depended on A and BofA (**S12 Fig**), supporting the idea that the F66A substitution did not disrupt interactions between the protease and its negative regulators. Collectively, these data are consistent with the model that the B protease adopts open and closed conformations *in vivo*.

## Discussion

Altogether, the results presented here are consistent with the substrate-gating model originally proposed by Shi and co-workers [30]. In the context of the pro-σ^K^ processing pathway, we envision that A and BofA inhibit the site-2 protease by stabilizing a closed, substrate-inaccessible conformation that prevents pro-σ^K^ access to the caged interior of the protease (**Fig 1A**). IVB-dependent (site-1) cleavage of A destabilizes this conformation leading to lateral displacement of the first and sixth transmembrane segments of the membrane-embedded protease allowing the pro-domain of σ^K^ access to the catalytic center of the enzyme. How cleavage of A triggers the open conformation is not known, but our data suggest that A need not be released from the complex as B(F66A)-YFP can process pro-σ^K^ while likely still bound to A and BofA and previous studies suggest that A remains tethered to the complex after IVB cleavage [22]. However, we note that pro-σ^K^ processing and σ^K^ activation are delayed in the B(F66A) mutant in the absence of IVB (**Fig 6D, S11A** and **S11B Fig**) and the localization of pro-σ^K^-CFP to the forespore membranes in a B(E44Q, F66A)-YFP strain still required the IVB signal, suggesting that the B(F66A) mutant samples both open and closed states with A and BofA promoting the closed conformation. Future experiments will be directed toward understanding how IVB-dependent cleavage of A destabilizes the closed conformation triggering lateral gate opening.

### What is the role of the CBS domain?

Reconstitution studies using recombinant B protease revealed that pro-σ^K^ processing requires ATP [13]. In line with this finding, the CBS domain of the B protease was shown to bind ATP *in vitro* [13]. Furthermore, as described above, a deletion of the C-terminal CBS domain did not support cleavage of a modified pro-σ^K^ substrate in an *E*. *coli* expression system [38]. Since CBS domains have been proposed to function as sensors of cellular energy status [31, 32] it was hypothesized that the CBS domain on the B protease might couple σ^K^ activation to the energy status of the mother cell. Here, we report that the CBS domain contributes to the association between B and pro-σ^K^ and the efficiency pro-σ^K^ processing in *B*. *subtilis* but is not strictly required for either nor is it necessary for efficient spore formation. We cannot account for the discrepancies between our *in vivo* analysis and the *in vitro* reconstitution and *E*. *coli* expression data reported previously, however, other differences between *B*. *subtilis* sporulation and these heterologous systems have been reported [38, 42]. *In vitro* studies with the purified CBS domain of B [13] and with the CBS module from the S2P from *Archaeoglobus fulgidus* [43] suggest that Mg^2+^-bound ATP favors monomerization. While it is possible that fusing YFP to BΔ66 influenced its ability to dimerize, we obtained similar results using a BΔ66 fusion to monomeric YFP and a YFP variant that retains the ability to dimerize (**S13 Fig**). Nonetheless, it is possible that replacing the CBS domain with YFP could mimic the activated conformation of this domain. We note that a BΔ66 truncation that lacks the YFP fusion is non-functional, however we do not have antibodies to the membrane protease domain to assess the stability of this mutant. That being said, we favor a deterministic model in which the activation of σ^K^ is principally governed by the developing spore via the production and secretion of the IVB signal and that mother cell energy status could dictate the efficiency of pro-σ^K^ processing and consequently the rate at which σ^K^-directed genes accumulate in the mother cell.

### RIP signaling during sporulation

The pro-σ^K^ processing pathway is distinct from the other characterized RIP signaling pathways in that the substrate of the intramembrane cleaving protease, pro-σ^K^, is not an integral membrane protein and is not subject to sequential site-1 and site-2 cleavages. Yet, this pathway takes advantage of a 2-step proteolytic cascade to transduce information across the lipid bilayer. We wonder whether other RIP signaling pathways that employ members of the group 3 S2P subfamily function similarly. *M*. *jannaschii* does not appear to have a homolog of the A protein. However, it does encode a protein with homology to BofA and a IVB protease family member despite not being an endospore-forming organism. It will be interesting to identify native substrates of mjS2P and other members of this broadly conserved subfamily. In the case of pro-σ^K^ pathway, this variation on RIP signaling is perfectly matched with the morphological constraints that exist during sporulation. B, A, and BofA are produced in the mother cell during the morphological process of engulfment [20, 21], while pro-σ^K^ expression is subject to more stringent control [44, 45] and only accumulates around the time when engulfment is complete [44, 46]. Due to a membrane fission event at this late stage [47], the mother-cell membranes that surround the forespore become topologically distinct from the peripheral membranes of the mother cell (**Fig 1A**) [48]. Integral membrane proteins produced in the mother cell at this stage are exclusively inserted in the peripheral membranes and are therefore unable to access the membranes surrounding the forespore [49]. Accordingly, to have access to the B protease, pro-σ^K^ must be a peripherally-associated, rather than an integral, membrane protein. Thus, regulation of intramembrane proteolysis is achieved by a site-1 signaling protease that instead cleaves a negative regulator of the S2P family member.

As in the case with RseP and other S2P family members, a complete picture of the sporulation RIP signaling pathway awaits structural determination of B(E44Q) bound to pro-σ^K^ and the inhibited B-A-BofA signaling complex. With the recent advances in membrane protein crystallography using lipidic cubic phase and cryo-electron microscopy, these structures are now within reach.

## Materials and methods

### General methods

All *B*. *subtilis* strains were derived from the prototrophic strain PY79 [50]. Sporulation was induced by resuspension at 37°C according to the method of Sterlini-Mandelstam [51] or by exhaustion in supplemented DS medium [52]. Sporulation efficiency was determined in 24–30 hour cultures as the total number of heat-resistant (80°C for 20 min) colony forming units (CFUs) compared with wild-type heat-resistant CFUs. All sporulation assays reported were based on three or more biological replicates. Expression of B, A, BofA and pro-σ^K^ during vegetative growth was performed in LB. IPTG was added to a final concentration of 0.5 mM at an OD_600_ of 0.1 and samples were analyzed every 30 minutes post induction. Deletion mutants were generated by isothermal assembly [53] and direct transformation into *B*. *subtilis*. Tables of strains (S1 Table), plasmids (S2 Table), oligonucleotide primers (S3 Table) and descriptions of plasmid construction and isothermal assembly deletion mutants can be found online as supplementary material.

### β-Galactosidase assays

Strains bearing the P_*gerE*_*-lacZ* fusion were sporulated by resuspension. 1 mL samples were collected by centrifugation every hour during sporulation and stored at -20 ˚C. Samples were processed by the method of Miller [51, 54], using *ortho*-nitrophenyl-β-D-galactopyranoside (ONPG) as substrate. β-Galactosidase specific activity was defined as the change in A_420_ per minute per milliliter of culture per OD_600_ × 1000 and is reported in Miller units. All β-Galactosidase assays reported were based on three or more biological replicates. σ^K^ activity during sporulation was also assessed on DSM agar plates containing 100 μg/mL 5-bromo-4-chloro-3-indolyl β-D-galactopyranoside (X-gal).

### Immunoblot analysis

Whole-cell lysates from sporulating cells (induced by resuspension) or vegetatively-growing cells were prepared as described previously [34, 55]. Equivalent loading was based on OD_600_ at the time of harvest. Proteins were separated by SDS-PAGE on 12.5% polyacrylamide gels, electroblotted onto Immobilon-P membranes (Millipore) and blocked in 5% nonfat milk in phosphate-buffered saline (PBS)-0.5% Tween-20. The blocked membranes were probed with anti-σ^K^ (1:10,000) [37], anti-σ^A^ (1:10,000) [56], anti-GFP (anti-10,000) [33], or anti-SpoIIP (1:10,000) [57] diluted into 3% BSA in 1X PBS-0.05% Tween-20. Primary antibodies were detected using horseradish peroxidase-conjugated goat, anti-rabbit IgG (1:20,000; BioRad) and the Western Lightning reagent kit as described by the manufacturer (PerkinElmer). At least two biological replicates were performed for each immunoblot. To resolve pro-σ^K^-CFP (55 kDa) and mature σ^K^-CFP (53 kDa), lysates were separated on a 35 cm 10% polyacrylamide gel.

### Fluorescence microscopy

Fluorescence microscopy was performed with an Olympus BX61 microscope as previously described [58]. Cells were mounted on a 2% agarose pad containing resuspension medium using a gene frame (BioRad). Fluorescent signals were visualized with a phase contrast objective UplanF1 100x and captured with a monochrome CoolSnapHQ digital camera (Photometrics) using MetaMorph software version 7.7 (Molecular devices). The membrane dye 1-(4-trimethylammoniumphenyl)-6-phenyl-1,3,5-hexatriene *p*-toluenesulfonate (TMA-DPH, Molecular Probes) was used at a final concentration of 50 μM and exposure times were typically 500 ms. The DNA dye 4’, 6-diamidino-2-phenylindole dihydrochloride (DAPI, Molecular Probes) was used at 2 μg/mL and exposure times were typically 200 ms. At least two biological replicates were performed for all microscopy experiment. Images were analyzed, adjusted and cropped using MetaMorph software.

### Immunoprecipitation and mass spectrometry

Immunoprecipitations were performed as described previously [59]. Briefly, 50 mL cultures were harvested at hour 4 after the initiation of sporulation by resuspension. Cell pellets were washed twice with 1X SMM (0.5 M sucrose, 20 mM MgCl_2_, 20 mM maleic acid pH 6.5) at room temperature and then resuspended in 5 mL 1X SMM containing lysozyme (0.5 mg/mL). The suspension was gently shaken for 30 minutes at room temperature to generate protoplasts. Protoplasts were collected by centrifugation and flash-frozen in N2(l). Thawed protoplasts were disrupted by osmotic lysis with 3 mL hypotonic buffer (Buffer H) (20 mM Hepes pH 8, 200 mM NaCl, 1 mM dithiothreitol, with protease inhibitors: 1 mM phenylmethylsulfonyl fluoride, 0.5 μg/mL leupeptin, 0.7 μg/mL pepstatin). MgCl_2_ and CaCl_2_ were added to 1 mM and lysates were treated with DNAse I (10 μg/mL final) and RnaseA (20 μg/mL) for 1 hour on ice. The membrane fraction was separated by ultracentrifugation at 35 krpm for 1 h at 4°C. The supernatant was removed, and the membrane pellet dispersed in 200 μL of Buffer G (Buffer H with 10% glycerol). Crude membrane preparations were aliquoted and flash-frozen in N_2_(l). 100 μL of crude membranes were diluted 5-fold with Buffer S (Buffer H with 20% glycerol and 100 μg/mL lysozyme), and membrane proteins were solubilized by the addition of the nonionic detergent digitonin (Sigma) to a final concentration of 0.5%. The mixture was rotated for 1 h at 4°C. Soluble and insoluble fractions were separated by centrifugation at 35 krpm for 1 h at 4°C. The soluble fraction (the load) was mixed with 20 μL of affinity-purified anti-GFP antibodies [22] covalently coupled to Protein A sepharose and rotated for 4 h at 4°C. The resin was pelleted at 5 krpm and washed five times with 1 mL of Buffer S + 0.5% digitonin. Immunoprecipitated proteins were eluted with 50 μL of sodium dodecyl sulfate (SDS) sample buffer (0.25 M Tris, pH 6.8, 6% SDS, 10 mM EDTA, 20% glycerol) and heated for 15 min at 50°C. The resin was pelleted and the supernatant (the IP) was transferred to a fresh tube and 2-mercaptoethanol was added to a final concentration of 10%. The immunoprecipitates were analyzed by immunoblot and SDS-PAGE followed by silver staining [59]. Individual bands were excised from silver-stained gels and trypsinized. Extracted peptides were then separated on a nanoscale C18 reverse-phase HPLC capillary column, and were subjected to electrospray ionization followed by MS using an LCQ DECA ion-trap mass spectrometer. Among the 5 peptides identified by MS, 3 (YLEILMAK, FGLDLKK, EIAKELGISR) were from σ^K^. No σ^K^ peptides were identified from the same region of the gel in the controls.

### Homology modeling

To generate homology models of the B protease, the sequence was first aligned to that of mjS2P using the HHPred server to generate an initial alignment for homology modeling [60]. This was further adjusted manually to correct a register error in the last transmembrane segment revealed by evolutionary co-variation analysis. The resulting modified alignment was used to construct a homology model in MODELLER [61] using the structures of mjS2P as a template (PDB ID: 3B4R). Both conformations were modeled, using 3B4R chain A as the template for the open conformation, and chain B as the template for the closed conformation.

### Generation of evolutionary couplings

Multiple sequence alignments (MSA) were generated for both the B protease (SP4FB_BACSU, residues 1–210) and mjS2P (Y392_METJA solved in PDB 3B4R, residues 1–224). The MSAs were built using jackhmmer [62], an iterative hidden Markov model-based sequence search tool, with 5 iterations querying against the April 2017 Uniref100 database [63]. For SP4FB_BACSU, the alignment contained 5,390 sequences (2,005 effective sequences after downweighting sequences with more than 80% identity), with 92.4% of the input residues covered with less than 30% gaps. Y392_METJA was aligned with 5,290 sequences (1927 effective sequences) with 92% coverage at 30% gap allowance. Evolutionary couplings were then determined as previously described [39, 64, 65]. The full EVFold package and documentation can be found at https://github.com/debbiemarkslab/EVcouplings

## Supporting information

S1 Methods Plasmids and strains construction. Details for plasmid and strain constructions in this study.
(PDF)

S1 Fig B-GFP resides in a membrane complex with A, BofA, SpoIIIAH (AH) and SpoIIQ (Q). **(A)** Silver-stained gel of immunoprecipitated proteins from detergent-solubilized membrane preparations of the indicated strains at hour 3.5 of sporulation. Proteins indicated on the right were identified by mass spectrometry or from immunoprecipitations using mutant strains. All lanes are from the same gel with unrelated lanes removed for clarity. (M, molecular weight markers in kDa). **(B)** Immunoblots from analogous immunoprecipitations. Digitonin-solubilized membrane preparation (L = Load), the supernatant after immunoprecipitation (FT = Flow Through), and the immunoprecipitate (IP) are shown. SpoIIQ (Q) and SpoIIIAH (AH) are more efficiently co-immunoprecipitated with GFP-A than B-GFP, consistent with cytological analysis suggesting AH and Q anchor A, while A in turn anchors B [35]. The EzrA immunoblot serves as a negative control.
(TIF)

S2 Fig B-YFP efficiently processes pro-σ^K^ and pro-σ^K^-CFP during sporulation. Immunoblots of the indicated strains monitoring pro-σ^K^ and pro-σ^K^-CFP processing during sporulation. **(A)** Time (in hours) after the initiation of sporulation is indicated above the blots. (top) B-YFP processes pro-σ^K^ to mature σ^K^ at similar stages and to a similar extent as wild-type B. SpoIIP is shown to control for loading. The proteins were separated by SDS-PAGE on 12.5% gels. The initiation of sporulation was delayed by ~45 minutes in these experiments compared to the ones shown in the cytological analysis. Because these cultures were sporulated side-by-side the timing of pro-σ^K^ and pro-σ^K^-CFP processing can be compared. **(B)** B-YFP but not B(E44Q)-YFP can process pro-σ^K^-CFP to mature σ^K^-CFP. The proteins were separated by SDS-PAGE on a 35 cm 10% gel. The blot was probed with anti-σ^K^ antibodies. Molecular weight markers (in kDa) are indicated to the left of the blots. **(C)** Analysis of liberated (free) CFP in cells expressing pro-σ^K^-CFP and B-YFP variants during vegetative growth. An anti-GFP immunoblot of the indicated strains is shown. The position of free CFP is highlighted with an asterisk. The lysates were heated at 80 ˚C for 10 minutes to denature YFP and CFP. The polytopic membrane protease B aggregates in SDS sample buffer above 50 ˚C. B-YFP aggregates are indicated. **(D)** Immunoblots from two independent sporulation time course experiments highlight the typical timing of pro-σ^K^ processing. B-YFP is shown for comparison. **(E)** Table of sporulation efficiencies of the indicated strains (*n* = 3).
(TIF)

S3 Fig Pro-σ^K^-CFP localization to the membranes surrounding the forespore requires IVB. Representative images of the indicated strains during a sporulation time course. Time (in hours) is indicated on the left. In the absence of IVB, pro-σ^K^-CFP localizes to the mother-cell cytoplasm with some enrichment on the nucleoid. Membranes were stained with the fluorescent dye TMA-DPH. Scale bar indicates 2 μm.
(TIF)

S4 Fig Quantification of forespore-localized pro-σ^K^-CFP. **(A)** Representative images of the four pro-σ^K^-CFP localization patterns that were quantified. These images were used for comparison during quantification. Sporulating cells in which pro-σ^K^-CFP localized adjacent to the forespore exclusively on the mother cell side was termed the "beard" pattern. We suspect this localization pattern is non-specific as it was principally observed in cells lacking the IVB signal or the B protease. **(B)** Quantification of forespore-localized pro-σ^K^-CFP in the indicated strains (from Figs 2 and S3) at hour 4 of sporulation. The number of sporulating cells scored is indicated below the bar graph. **(C)** Quantification of forespore-localized pro-σ^K^-CFP in the indicated strains (from Figs 3 and S5) at hour 4 of sporulation. **(D)** Quantification of forespore-localized pro-σ^K^-CFP in the indicated strains (from Figs 5 and S7) at hour 4.5 of sporulation.
(TIF)

S5 Fig **Pro-σ^K^-CFP localization to the membranes surrounding the forespore requires the B protease but not A or BofA.** Representative images of the indicated strains during a sporulation time course. Time (in hours) is indicated above each set of strains. Pro-σ^K^-CFP co-localizes with B(E44Q)-YFP in the mother-cell membranes surrounding the forespore in cells harboring the IVB signaling protease. In sporulating cells lacking the B protease, pro-σ^K^-CFP localizes to the mother cell cytoplasm with some enrichment on the nucleoid. In the absence of A, BofA or both, pro-σ^K^-CFP localizes around the forespore. The cytoplasmic pro-σ^K^-CFP signal is more pixelated in these strains compared to the Δ*B* mutant. Membranes were stained with the fluorescent dye TMA-DPH. Images were scaled identically. Scale bar indicates 2 μm.
(TIF)

S6 Fig Pro-σ^K^-CFP localizes to the membranes of vegetatively growing cells when B(E44Q) is co-expressed. Representative images of the indicated strains during an induction time course. Pro-σ^K^-CFP, B-YFP, and B(E44Q)-YFP were expressed under IPTG control. Time in minutes after IPTG addition is indicated. Pro-σ^K^-CFP localizes in the cytoplasm in the absence of B and to nucleoid when wild-type B-YFP is co-expressed. Pro-σ^K^-CFP co-localizes with B(E44Q)-YFP in the septal and cytoplasmic membranes when co-expressed. The cytoplasmic pro-σ^K^-CFP signal is weaker and more pixelated in the strain expressing B(E44Q)-YFP compared to the one lacking B. All images were scaled identically. Scale bar indicates 2 μm.
(TIF)

S7 Fig The CBS domain of the B protease is not critical for pro-σ^K^-CFP localization. **(A)** Representative images of the indicated strains during a sporulation time course. Time (in hours) is indicated above each set of strains. Pro-σ^K^-CFP localizes around the forespore in otherwise wild-type cells with a full-length CBS domain or cells harboring 10 or 66 amino acid C-terminal truncations of the B protease. Pro-σ^K^-CFP localizes to the mother-cell cytoplasm with some enrichment on the nucleoid in the presence of a larger 85 amino acid truncation of B, similar to sporulating cells lacking the B protease. All images were scaled identically. Scale bar indicates 2 μm. **(B)** Quantification of forespore-associated B-YFP fluorescence in the indicated strains. ImageJ was used to measure the background-subtracted YFP pixel intensity associated with the forespore. 100 sporulating cells were analyzed per strain.
(TIF)

S8 Fig Pro-σ^K^-CFP localization around the forespore in the absence of the CBS domain on B requires IVB signaling. Representative images of the indicated strains at hour 4.5 of sporulation. Pro-σ^K^-CFP localizes around the forespore in cells harboring BΔ66-YFP provided the IVB signal is produced. All images were scaled identically. Yellow carets highlight the localization of pro-σ^K^-CFP around the forespore. Scale bar indicates 2 μm.
(TIF)

S9 Fig The CBS domain on B is not required for IVB-dependent σ^K^ activity and pro-σ^K^ processing. **(A)** σ^K^ activity was monitored using a σ^K^-responsive promoter (P_*gerE*_) fused to *lacZ*. β-galactosidase activity was determined during a sporulation time course in the indicated strains (*n* = 3). Time (in hours) after the initiation of sporulation is indicated. In the presence of IVB, σ^K^ activity is induced with similar kinetics to wild-type in strains harboring wild-type B-YFP, B(Δ10)-YFP, and B(Δ66)-YFP but not B(Δ85)-YFP. In the absence of IVB, σ^K^ is not significantly activated. **(B)** Immunoblot of the indicated strains monitoring pro-σ^K^ processing during a sporulation time course. Time (in hours) after the initiation of sporulation is indicated above the blots. (top) B(Δ66)-YFP processes pro-σ^K^ to mature σ^K^ at a similar stage and to a similar extent as wild-type but slightly delayed compared to a match wild-type control (B-YFP). (bottom) SpoIIP is shown to control for loading. The initiation of sporulation was delayed by ~45 minutes in these experiments compared to the ones used for cytological analysis, however because these cultures were sporulated side-by-side the timing of σ^K^ activity and pro-σ^K^ processing can be compared.
(TIF)

S10 Fig Co-variation analysis of mjS2P and B. **(A)** Interaction maps showing evolutionary couplings (ECs) (black dots) compared to true contacts (blue dots) (<5 Å) based on the crystal structure of mjS2P (PDB 3B4R) in the open (chain A) and closed (chain B) conformations. Orange dots show interactions (<5 Å) between the two mjS2P molecules in the asymmetric unit of the crystal that form an anti-parallel pseudodimer. ECs shown (86 total) were determined at a probability threshold of 90%. **(B)** Co-variation analysis of the B protease. Interaction map showing 81 ECs (black dots) at a probability threshold of 90%. Blue dots indicate residue contacts within 5 Å of each other, determined from the homology models in the open and closed conformations. Axes represent sequence indices.
(TIF)

S11 Fig B(F66A) activates σ^K^ in the absence of site-1 signaling proteases. **(A)** σ^K^ activity was monitored using a σ^K^-responsive promoter (P_*gerE*_) fused to *lacZ*. Liquid β-galactosidase assay during a sporulation time course in the indicated strains (*n* = 3). Only B(F66A)-YFP activates σ^K^ in the absence of IVB (open triangles) and in the absence of both IVB and CtpB (open squares). The initiation of sporulation was delayed by ~45 minutes in these experiments compared to the ones used for cytological analysis, however because these cultures were sporulated side-by-side the timing of σ^K^ activity can be compared. **(B)** Immunoblot analysis of pro-σ^K^ processing in the same strains used in (A). Time (in hours) after the initiation of sporulation is shown above the blot. SpoIIP immunoblot is shown to control for loading. **(C)** Sporulation efficiency is modestly reduced in cells lacking BofA or harboring B(F66A). Sporulation efficiencies of the indicated strains (*n* = 3) are shown.
(TIF)

S12 Fig B(F66A)-YFP localization to the membranes surrounding the forespore requires A and BofA. Representative images of the indicated strains at hour 4.5 of sporulation. B(F66A)-YFP localizes to the membranes surrounding the forespore in the presence and absence of the IVB protease **(A)** but not in sporulating cells lacking A or BofA **(B).** To more easily compare B-YFP signal in the mother-cell peripheral membranes the images were re-scaled to reveal weaker signals (pushed signal). Membranes were stained with the fluorescent dye TMA-DPH. Images from each strain were scaled identically. Scale bar indicate 2 μm.
(TIF)

S13 Fig The BΔ66 mutant behaves identically regardless of whether it is fused to mYFP or a YFP variant that is capable of dimerization. **(A)** Sporulation efficiency of the indicated strains (*n* = 3). **(B)** σ^K^ activity was monitored using a σ^K^-responsive promoter (P_*gerE*_) fused to *lacZ*. Image of a sporulation agar plate containing X-gal with the indicated strains after 24 hours of incubation at 37˚C. **(C)** Representative images of the indicated strains at hour 4.5 of sporulation. Localization of pro-σ^K^-CFP around the forespore (yellow carets) in strains with an intact IVB (site-1) signaling protease is indicated. Membranes were stained with the fluorescent dye TMA-DPH. Images were scaled identically. Scale bar indicates 2 μm.
(TIF)

S1 Table *Bacillus subtilis* strains used in this study. All strains, their genotypes, and sources are listed in this table.
(PDF)

S2 Table Plasmids used in this study. All plasmids and their sources are listed in this table.
(PDF)

S3 Table Oligonucleotide primers used in this study. All oligonucleotides used for plasmid construction, gene deletion, or sequencing are listed in this table. Capital letters were used for restriction endonuclease recognition sites and underlined letters indicate mutated bases.
(PDF)
