# Supplementary material for: Evidence that regulation of intramembrane proteolysis is mediated by substrate gating during sporulation in Bacillus subtilis
Source: PLoS Genet. 2018 Nov 7;14(11):e1007753. doi: 10.1371/journal.pgen.1007753 (PMC6242693; doi:10.1371/journal.pgen.1007753)
Supplement: S1 Methods — Details for plasmid and strain constructions in this study. (PDF) [file pgen.1007753.s001.pdf]

SUPPLEMENTAL INFORMATION

Evidence that regulation of intramembrane proteolysis is mediated by substrate gating during sporulation in *Bacillus subtilis*

Fernando H. Ramírez-Guadiana, Christopher D. A. Rodrigues, Kathleen A. Marquis, Nathalie Campo, Rocío del Carmen Barajas-Ornelas, Kelly Brock, Debora S. Marks, Andrew C. Kruse, David Z. Rudner

This PDF file includes:

Supplemental Methods

S1 Methods. Plasmids and strains construction.

pKM190 [*spoIVFB-yfp* (*spec*)] (single crossover integration) was generated in a two-way ligation with an MfeI-XhoI PCR product containing the 3' end of the *spoIVFB* gene (oligonucleotide primers oDR481 + oDR482 and pDR004 (*spoIVF* operon) DNA as template) and pKL183 cut with *Eco*RI and *Xho*I. pKL183 (Lemon & Grossman 2000) is a single crossover integration vector containing the *yfp* gene.

pKM261 [*ycgO::PspoIVF-spoIVFB(E44Q)-yfp (erm)*] was generated in a three-way ligation with a *Hind*III-*Xho*I PCR product containing spoIVFB(E44Q) (oligonucleotide primers oDR106 + oDR482 and pDR019 DNA as template), a *Xho*I-*Bam*HI PCR product contain the *yfp* gene (oligonucleotide primers oDR78 + oDR79 and pKL183 as template), and pKM259 cut with *Hind*III and *Bam*HI. pKM259 [*ycgO*::P*spoIVF* (erm)] was generated in a two-way ligation with an *EcoRI-Hind*III fragment containing the PspoIVF promoter from pDR077 and pKM084 cut with *Eco*RI and *Hind*III. pKM084 [*ycgO::erm*] is an ectopic integration vector for double crossover integration at the ycgO locus (KAM and DZR, unpublished)

pKM266 [*spoIIIC-cfp (cat)*] (single crossover integration) was generated in a two-way ligation with an *Eco*RI-*Xho*I PCR product containing the 3' end of the spoIIIC (oligonucleotide primers oDR594 + oDR595 and pSK6 DNA as template) and pKM135 cut with *Eco*RI and *Xho*I. pKM135 is a single crossover integration vector containing the *mCFP* gene.

pKM283 [*ycgO::PspoIVF-spoIVFB-yfp (erm)*] was generated in a two-way ligation with a *Hind*III-*Xho*I PCR product containin the spoIVFB gene (oligonucleotide primers oDR106 + oDR482 and pDR004 DNA as template) and pKM261 cut with *Hind*III and *Xho*I.

pCR275 [*ycgO::Pspank-spoIVFB(E44Q)-yfp (erm)*] was generated in a two-way ligation with a *Hind*III-*Nhe*I PCR product containing *spoIVFB(E44Q)-yfp* (oligonucleotide primers oCR603 + oCR604 and pKM261 DNA as template) and pER65 cut with *Hind*III-*Nhe*I. pER65 [*ycgO::Pspank (lacI) (erm)*] is a double crossover vector, with an IPTG-inducible promoter, for ectopic integration at the *ycgO* locus (E. Riley and DZR, unpublished).

pCR276 [*ycgO::Pspank-spoIVFB-yfp (erm)*] was generated in a two-way ligation with a *Hind*III-*Nhe*I PCR product containing *spoIVFB-yfp* (oligonucleotide primers oCR603 + oCR604 and pKM283 DNA as template) and pER065 cut with *Hind*III-*Nhe*I.

pCR278 [*amyE::Pspank-pro-sigK-cfp (spec)*] was generated in a three-way ligation with a *Hind*III-*Nhe*I PCR product containing *pro-sigK* (oligonucleotide primers oCR599 + oCR600 and pDR012 (*amyE::pro-sigK (cat)*) DNA as template), an *Nhe*I-*Sph*I PCR product containing m*cfp* (oligonucleotide primers oCR601 + oCR606 and pCR034 (*sacA::PspoIIQ-cfp-spoIIQ (spec)*) DNA as template), and pDR110 cut with *Hind*III and *Sph*I. pDR110 [*amyE*::*Pspank (lacI) (spec)*] is a

double crossover vector, with an IPTG-inducible promoter, for ectopic integration at the *amyE* locus (DZR, unpublished).

pCR286 [*ycgO::PspoIVF-spoIVFB(E44Q)Δ10-yfp (erm)*] was generated in a two-way ligation with *Hind*III-*Xho*I PCR product containing *spoIVFB(E44Q)Δ10* (oligonucleotide primers oDR106 + oCR619 on pKM261 DNA as template) and pKM261 cut with *Hind*III-*Xho*I.

pCR287 [*ycgO::PspoIVF-spoIVFB(E44Q)Δ66-yfp (erm)*] was generated in a two-way ligation with *Hind*III-*Xho*I PCR product containing *spoIVFB(E44Q)Δ66* (oligonucleotide primers oDR106 + oCR620 and pKM261 DNA as template) and pKM261 cut with *Hind*III-*Xho*I.

pCR288 [*ycgO::PspoIVF-spoIVFB(E44Q)Δ85-yfp (erm)*] was generated in a two-way ligation with a *Hind*III-*Xho*I PCR product containing *spoIVFB(E44Q)Δ85* (oligonucleotide primers oDR106 + oCR621 and pKM261 DNA as template and pKM261 cut with *Hind*III-*Xho*I.

pFR20 [*ycgO*::*PspoIVF-spoIVFB*Δ*10-yfp (erm)*] was generated in a two-way ligation with a *Hind*III-*Xho*I PCR product containing *spoIVFB*Δ10 (oligonucleotide primers oDR106 + oCR619 and genomic DNA from PY79 as template) and pKM261 cut with *Hind*III-*Xho*I.

pFR21 [*ycgO*::*PspoIVF-spoIVFB*Δ*66-yfp (erm)*] was generated in a two-way ligation with a *Hind*III-*Xho*I PCR product containing *spoIVFB*Δ66 (oligonucleotide primers oDR106 + oCR620 and genomic DNA from PY79 as template) and pKM261 cut with *Hind*III-*Xho*I.

pFR22 [*ycgO*::*PspoIVF-spoIVFB*Δ*85-yfp (erm)*] was generated in a two-way ligation with a *Hind*III-*Xho*I PCR product containing *spoIVFB*Δ85 (oligonucleotide primers oDR106 + oCR621 and genomic DNA from PY79 as template) and pKM261 cut with *Hind*III-*Xho*I.

pFR28 [*yvbJ::Phyperspank-spoIVFA* (*cat*)] was generated by isothermal assembly of two pieces: 1) plasmid pMS024 cut with HindIII and NheI; 2) a PCR product containing *spoIVFA* (oligonucleotide primers oFR58 + oFR59 and genomic DNA from PY79 as template). pMS024 [*yvbJ::Phyperspank (cat)*] is an ectopic integration vector harboring the P*xylA* promoter for double crossover integrations at the *yhdG* locus (M. Stanley and DZR, unpublished).

pFR29 [*yhdG::Phyperspank-bofA* (*kan*)] was generated in a two-way ligation with a *Spe*I-*Sph*I PCR product containing *bofA* (oligonucleotide primers oFR62 + oFR66 and genomic DNA from PY79 as template) and pMS036 cut with *Spe*I and *Sph*I. pMS036 [*yhdG::Phyperspank (kan)*] is an ectopic integration vector harboring the P*hyperspank* promoter for double crossover integrations at the *yhdG* locus (M. Stanley and DZR, unpublished).

pFR30 [*lacA*::*spoIVB*(S378A) (*tet*)] was generated in a two-way ligation with an *Xho*I-*Nhe*I PCR product containing *spoIVB*(S378A) (oligonucleotide primers oFR72 + oFR73 and genomic DNA

from BDR1454) and pNC018 cut with *Xho*I and *Nhe*I. pNC018 [*lacA::tet*] is an ectopic integration vector for double crossover integrations at the *lacA* locus (NC and DZR, unpublished).

pFR31 [*ycgO*::*PspoIVF-spoIVFB(E44Q)Δ66*-m*yfp (erm)*] was generated in a two-way ligation with a *Xho*I-*Bam*HI PCR product containing *myfp* (oligonucleotide primers oFR77 + oFR78 and plasmid DNA from pKM012 as template) and pCR287 cut with *Xho*I-*Bam*HI. pKM012 contains the m*yfp* with codons optimized for *Bacillus subtilis* (KM and DZR, unpublished).

pFR32 [*ycgO*::*PspoIVF-spoIVFBΔ66-myfp (erm)*] was generated in a two-way ligation with a *Xho*I-*Bam*HI PCR product containing *myfp* (oligonucleotide primers oFR77 + oFR78 and plasmid DNA from pKM012 as template) and pFR21 cut with *Xho*I-*Bam*HI.

pFR36 [*ycgO*::*PspoIVF-spoIVFBΔ66 (erm)*] was generated in a two-way ligation with a *Hind*III-*Bam*HI PCR product containing *spoIVFB*Δ66 (oligonucleotide primers oFR83 + oFR84 and genomic DNA from PY79 as template) and pKM283 cut with *Xho*I-*Bam*HI.

pCB061 [*ycgO*::*PspoIVF-spoIVFB(F66A)-yfp (erm)*] was generated by site-directed mutagenesis oligonucleotide primers oCB038 + oCB039 and plasmid pKM283.

Strain construction:

BDR3685 [Δ*spoIVB::kan*] was generated by direct transformation of *B. subtilis* PY79 with an isothermal assembly product derived from 3 PCR products: 1) a PCR product containing an upstream region of *spoIVB* amplified with oligonucleotide primers oFR48 and oFR49 and *B. subtilis* PY79 genomic DNA as template; 2) a PCR product containing the Kan cassette; 3) a PCR product containing a downstream region of *spoIVB* amplified with oligonucleotide primers oFR50 and oFR51 and *B. subtilis* PY79 genomic DNA as template.
