# Supplementary figures and images for: Evidence that regulation of intramembrane proteolysis is mediated by substrate gating during sporulation in Bacillus subtilis
Source: PLoS Genet. 2018 Nov 7;14(11):e1007753. doi: 10.1371/journal.pgen.1007753 (PMC6242693; doi:10.1371/journal.pgen.1007753)

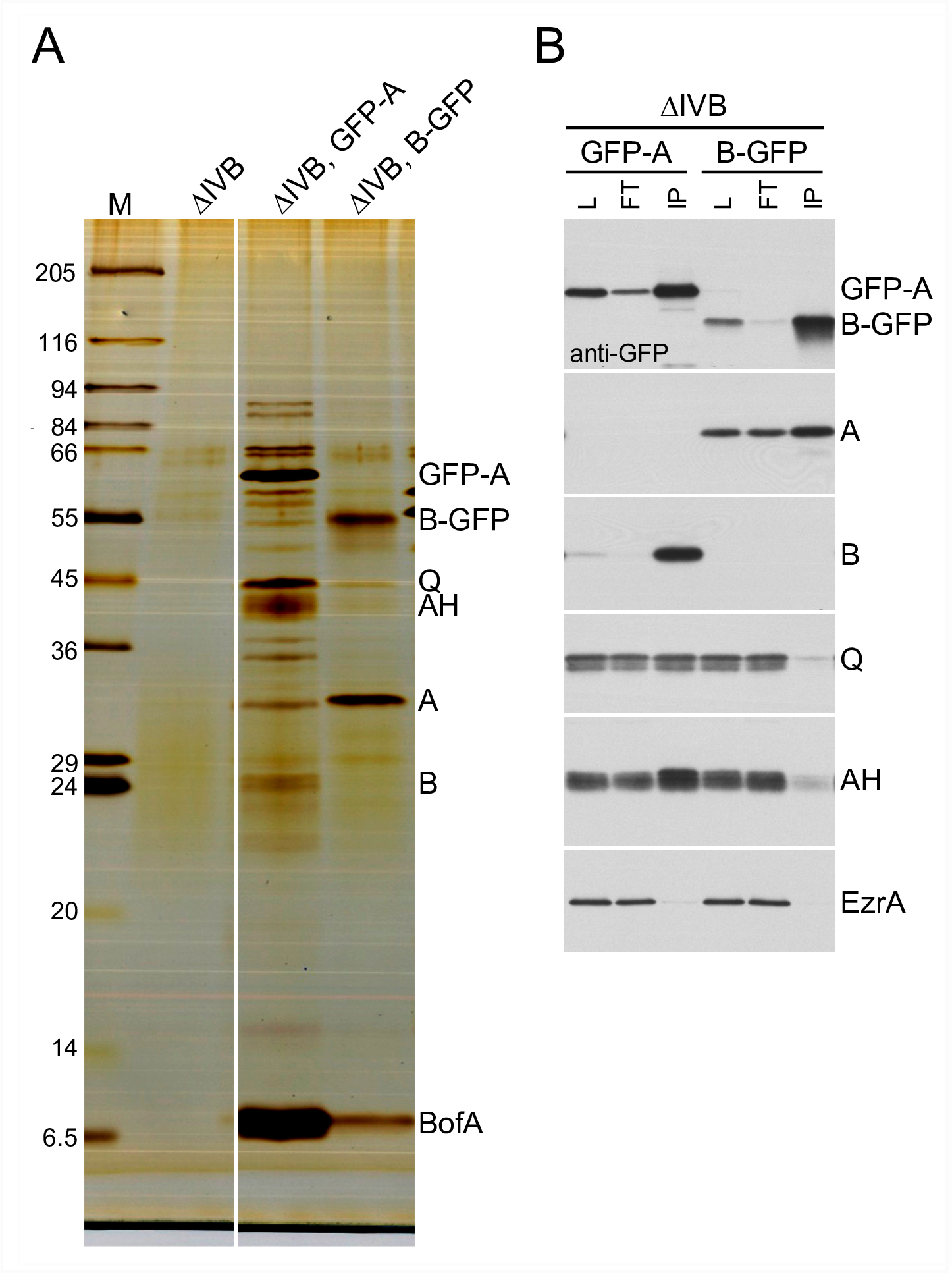

Supplement: S1 Fig — (A) Silver-stained gel of immunoprecipitated proteins from detergent-solubilized membrane preparations of the indicated strains at hour 3.5 of sporulation. Proteins indicated on the right were identified by mass spectrometry or from immunoprecipitations using mutant strains. All lanes are from the same gel with unrelated lanes removed for clarity. (M, molecular weight markers in kDa). (B) Immunoblots from analogous immunoprecipitations. Digitonin-solubilized membrane preparation (L = Load), the supernatant after immunoprecipitation (FT = Flow Through), and the immunoprecipitate (IP) are shown. SpoIIQ (Q) and SpoIIIAH (AH) are more efficiently co-immunoprecipitated with GFP-A than B-GFP, consistent with cytological analysis suggesting AH and Q anchor A, while A in turn anchors B [35]. The EzrA immunoblot serves as a negative control. (TIF) [file pgen.1007753.s002.tif]

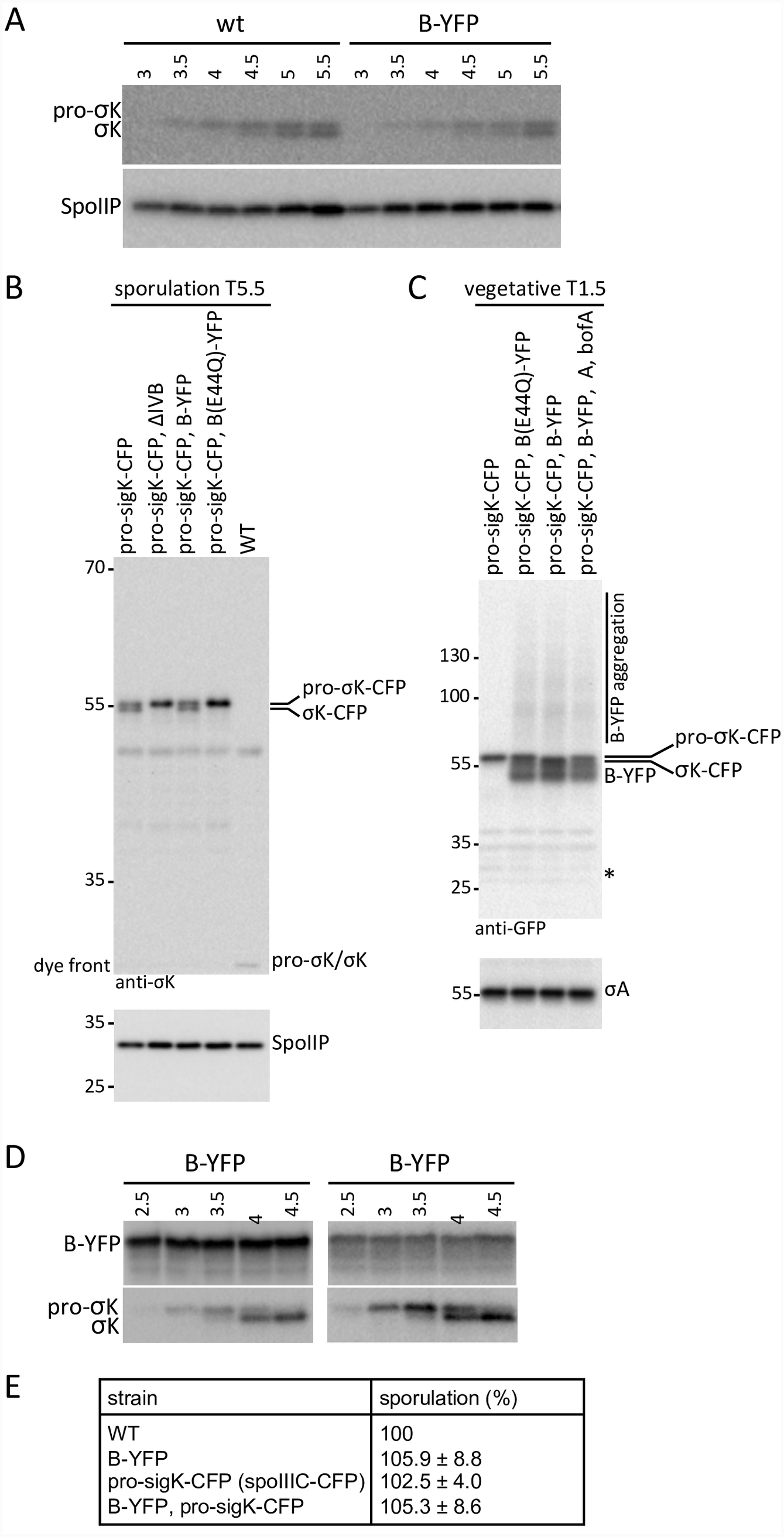

Supplement: S2 Fig — Immunoblots of the indicated strains monitoring pro-σK and pro-σK-CFP processing during sporulation. (A) Time (in hours) after the initiation of sporulation is indicated above the blots. (top) B-YFP processes pro-σK to mature σK at similar stages and to a similar extent as wild-type B. SpoIIP is shown to control for loading. The proteins were separated by SDS-PAGE on 12.5% gels. The initiation of sporulation was delayed by ~45 minutes in these experiments compared to the ones shown in the cytological analysis. Because these cultures were sporulated side-by-side the timing of pro-σK and pro-σK-CFP processing can be compared. (B) B-YFP but not B(E44Q)-YFP can process pro-σK-CFP to mature σK-CFP. The proteins were separated by SDS-PAGE on a 35 cm 10% gel. The blot was probed with anti-σK antibodies. Molecular weight markers (in kDa) are indicated to the left of the blots. (C) Analysis of liberated (free) CFP in cells expressing pro-σK-CFP and B-YFP variants during vegetative growth. An anti-GFP immunoblot of the indicated strains is shown. The position of free CFP is highlighted with an asterisk. The lysates were heated at 80 ˚C for 10 minutes to denature YFP and CFP. The polytopic membrane protease B aggregates in SDS sample buffer above 50 ˚C. B-YFP aggregates are indicated. (D) Immunoblots from two independent sporulation time course experiments highlight the typical timing of pro-σK processing. B-YFP is shown for comparison. (E) Table of sporulation efficiencies of the indicated strains (n = 3). (TIF) [file pgen.1007753.s003.tif]

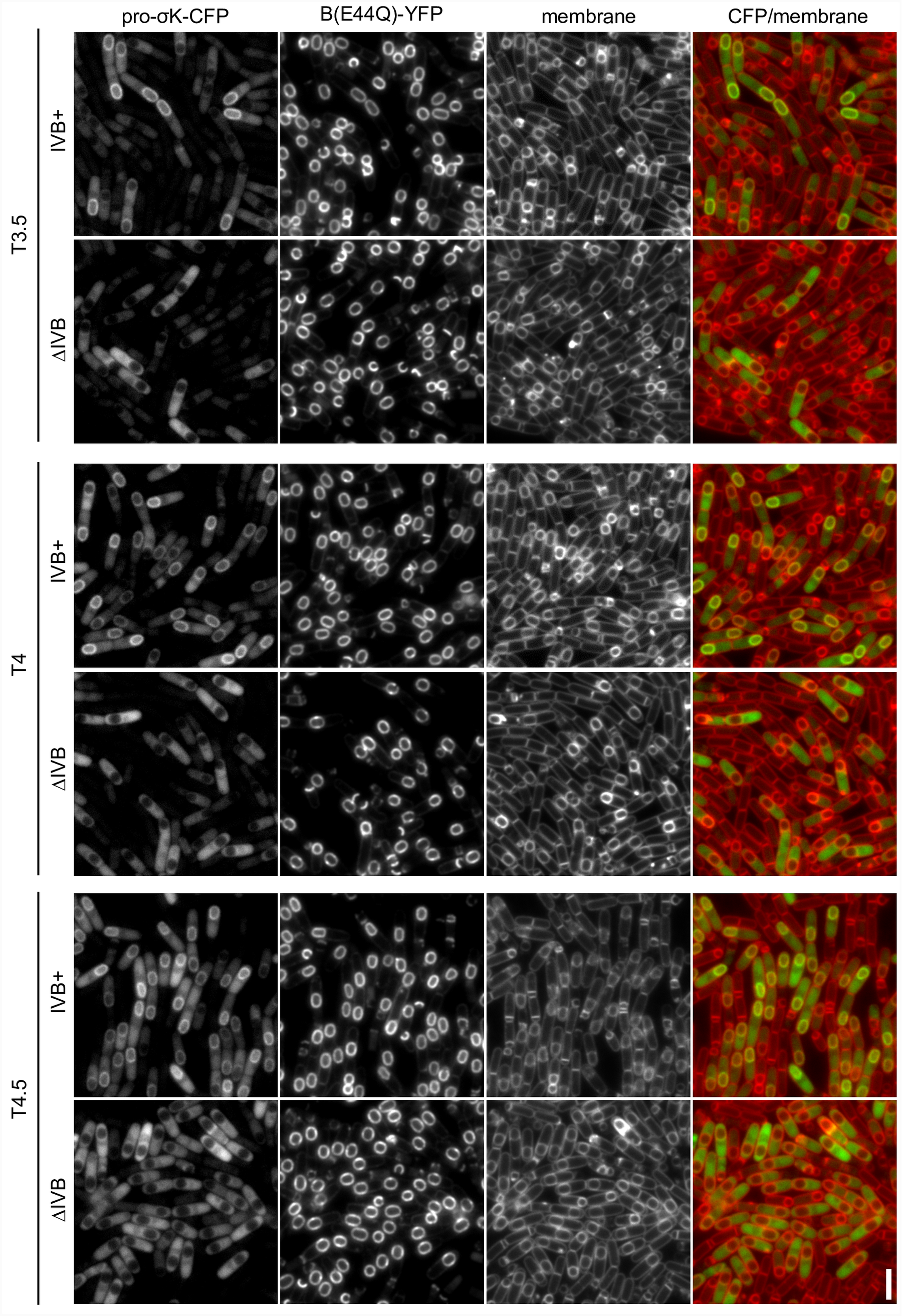

Supplement: S3 Fig — Representative images of the indicated strains during a sporulation time course. Time (in hours) is indicated on the left. In the absence of IVB, pro-σK-CFP localizes to the mother-cell cytoplasm with some enrichment on the nucleoid. Membranes were stained with the fluorescent dye TMA-DPH. Scale bar indicates 2 μm. (TIF) [file pgen.1007753.s004.tif]

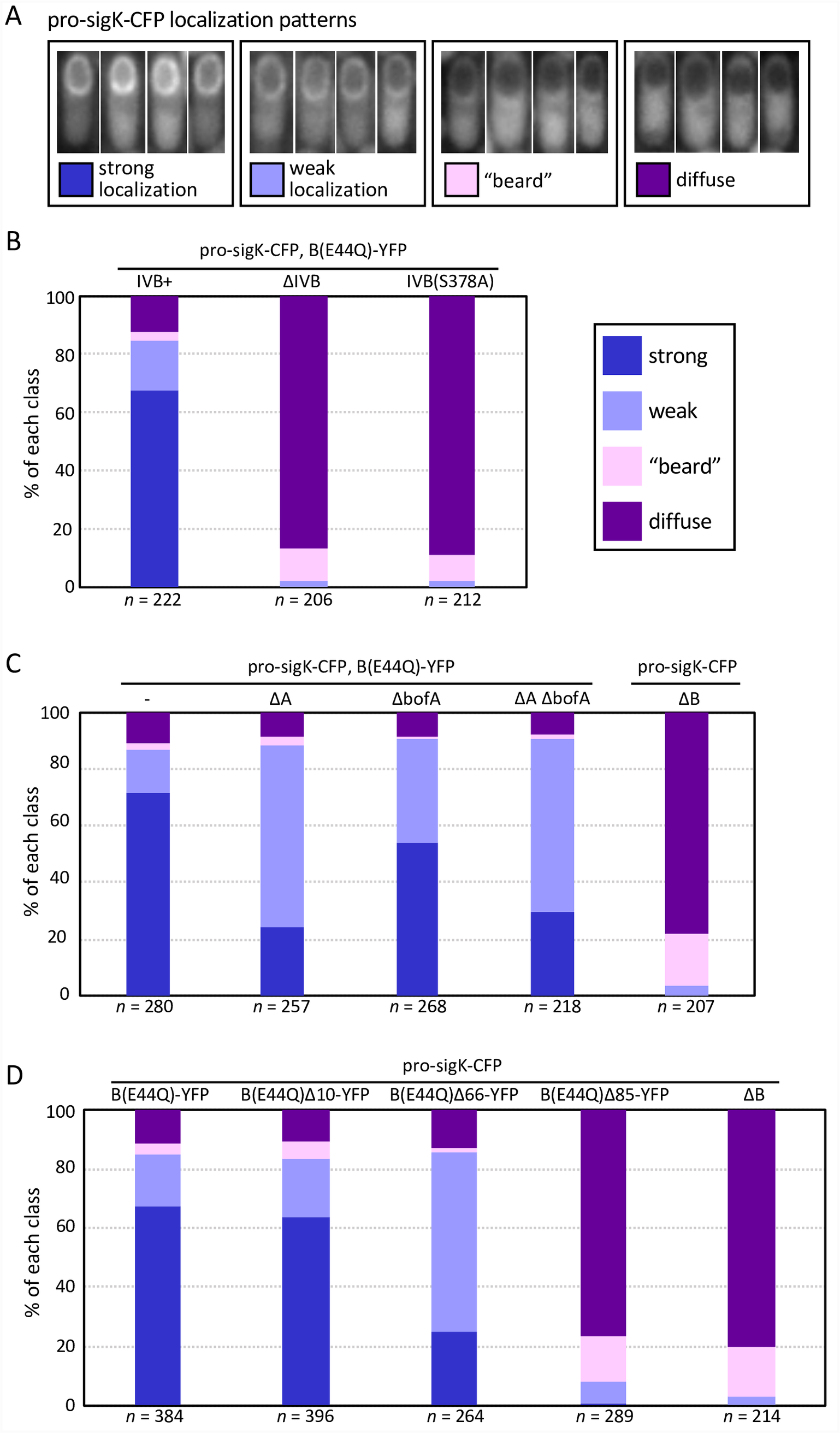

Supplement: S4 Fig — (A) Representative images of the four pro-σK-CFP localization patterns that were quantified. These images were used for comparison during quantification. Sporulating cells in which pro-σK-CFP localized adjacent to the forespore exclusively on the mother cell side was termed the "beard" pattern. We suspect this localization pattern is non-specific as it was principally observed in cells lacking the IVB signal or the B protease. (B) Quantification of forespore-localized pro-σK-CFP in the indicated strains (from Figs 2 and S3) at hour 4 of sporulation. The number of sporulating cells scored is indicated below the bar graph. (C) Quantification of forespore-localized pro-σK-CFP in the indicated strains (from Figs 3 and S5) at hour 4 of sporulation. (D) Quantification of forespore-localized pro-σK-CFP in the indicated strains (from Figs 5 and S7) at hour 4.5 of sporulation. (TIF) [file pgen.1007753.s005.tif]

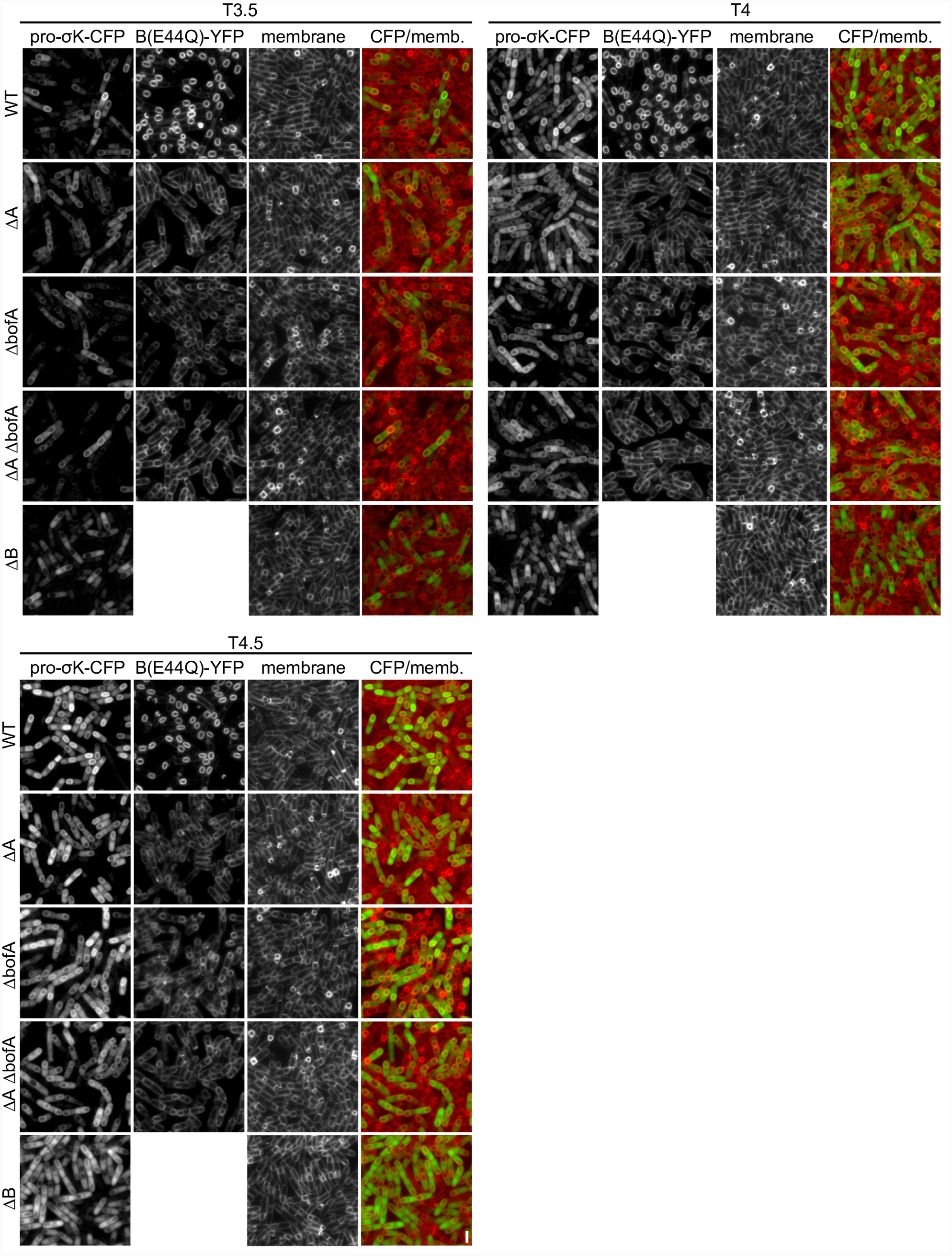

Supplement: S5 Fig — Pro-σK-CFP localization to the membranes surrounding the forespore requires the B protease but not A or BofA. Representative images of the indicated strains during a sporulation time course. Time (in hours) is indicated above each set of strains. Pro-σK-CFP co-localizes with B(E44Q)-YFP in the mother-cell membranes surrounding the forespore in cells harboring the IVB signaling protease. In sporulating cells lacking the B protease, pro-σK-CFP localizes to the mother cell cytoplasm with some enrichment on the nucleoid. In the absence of A, BofA or both, pro-σK-CFP localizes around the forespore. The cytoplasmic pro-σK-CFP signal is more pixelated in these strains compared to the ΔB mutant. Membranes were stained with the fluorescent dye TMA-DPH. Images were scaled identically. Scale bar indicates 2 μm. (TIF) [file pgen.1007753.s006.tif]

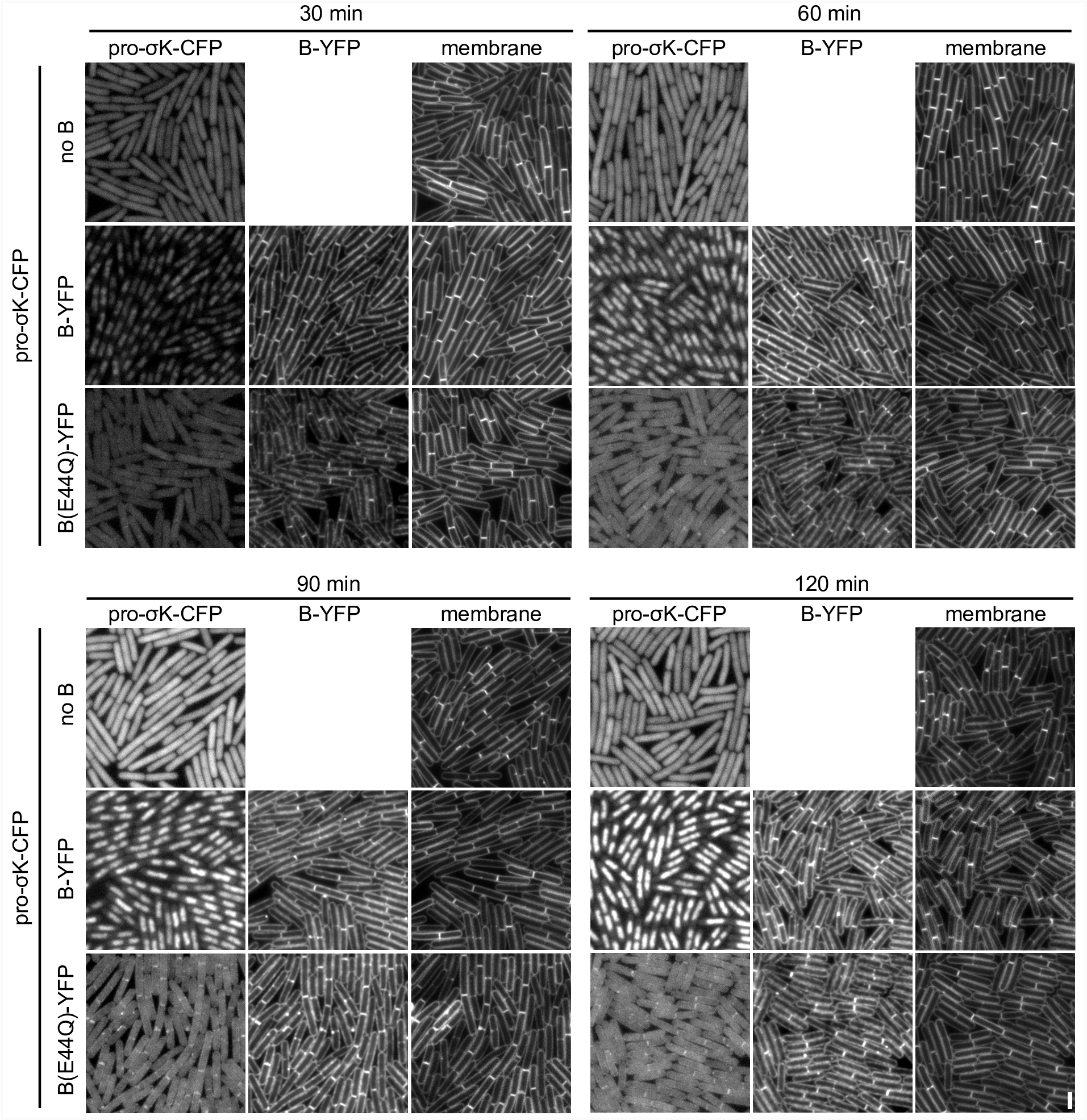

Supplement: S6 Fig — Representative images of the indicated strains during an induction time course. Pro-σK-CFP, B-YFP, and B(E44Q)-YFP were expressed under IPTG control. Time in minutes after IPTG addition is indicated. Pro-σK-CFP localizes in the cytoplasm in the absence of B and to nucleoid when wild-type B-YFP is co-expressed. Pro-σK-CFP co-localizes with B(E44Q)-YFP in the septal and cytoplasmic membranes when co-expressed. The cytoplasmic pro-σK-CFP signal is weaker and more pixelated in the strain expressing B(E44Q)-YFP compared to the one lacking B. All images were scaled identically. Scale bar indicates 2 μm. (TIF) [file pgen.1007753.s007.tif]

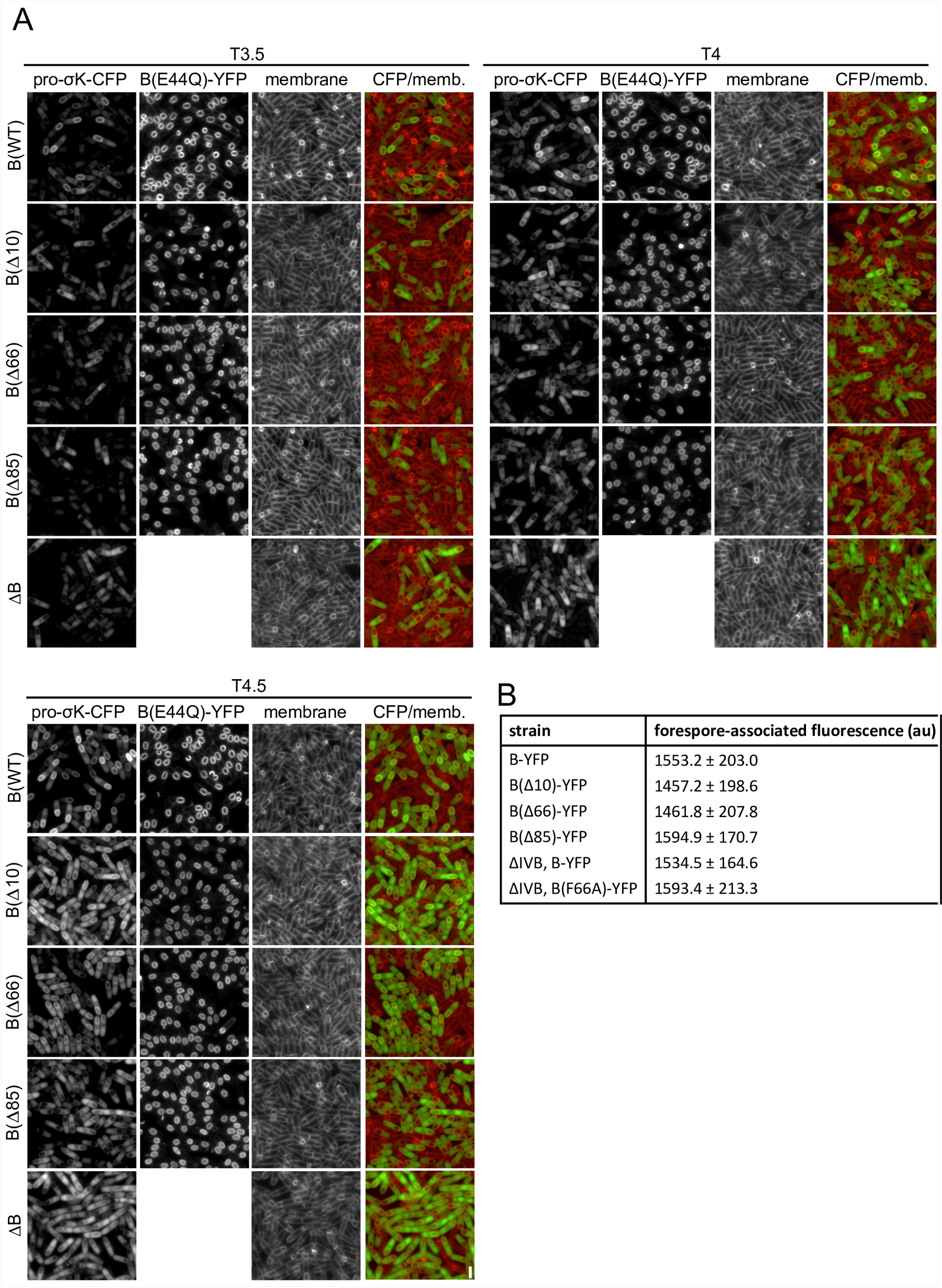

Supplement: S7 Fig — (A) Representative images of the indicated strains during a sporulation time course. Time (in hours) is indicated above each set of strains. Pro-σK-CFP localizes around the forespore in otherwise wild-type cells with a full-length CBS domain or cells harboring 10 or 66 amino acid C-terminal truncations of the B protease. Pro-σK-CFP localizes to the mother-cell cytoplasm with some enrichment on the nucleoid in the presence of a larger 85 amino acid truncation of B, similar to sporulating cells lacking the B protease. All images were scaled identically. Scale bar indicates 2 μm. (B) Quantification of forespore-associated B-YFP fluorescence in the indicated strains. ImageJ was used to measure the background-subtracted YFP pixel intensity associated with the forespore. 100 sporulating cells were analyzed per strain. (TIF) [file pgen.1007753.s008.tif]

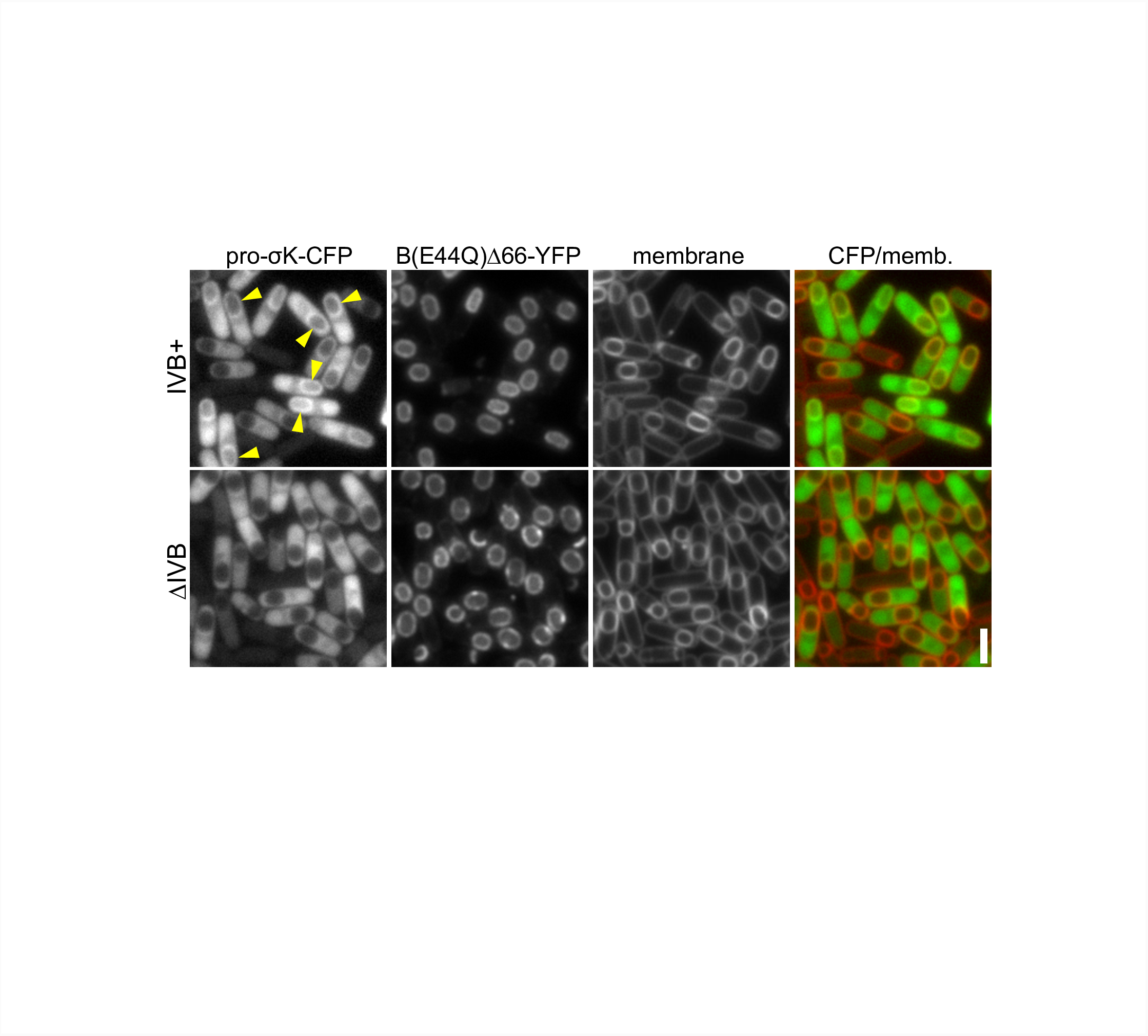

Supplement: S8 Fig — Representative images of the indicated strains at hour 4.5 of sporulation. Pro-σK-CFP localizes around the forespore in cells harboring BΔ66-YFP provided the IVB signal is produced. All images were scaled identically. Yellow carets highlight the localization of pro-σK-CFP around the forespore. Scale bar indicates 2 μm. (TIF) [file pgen.1007753.s009.tif]

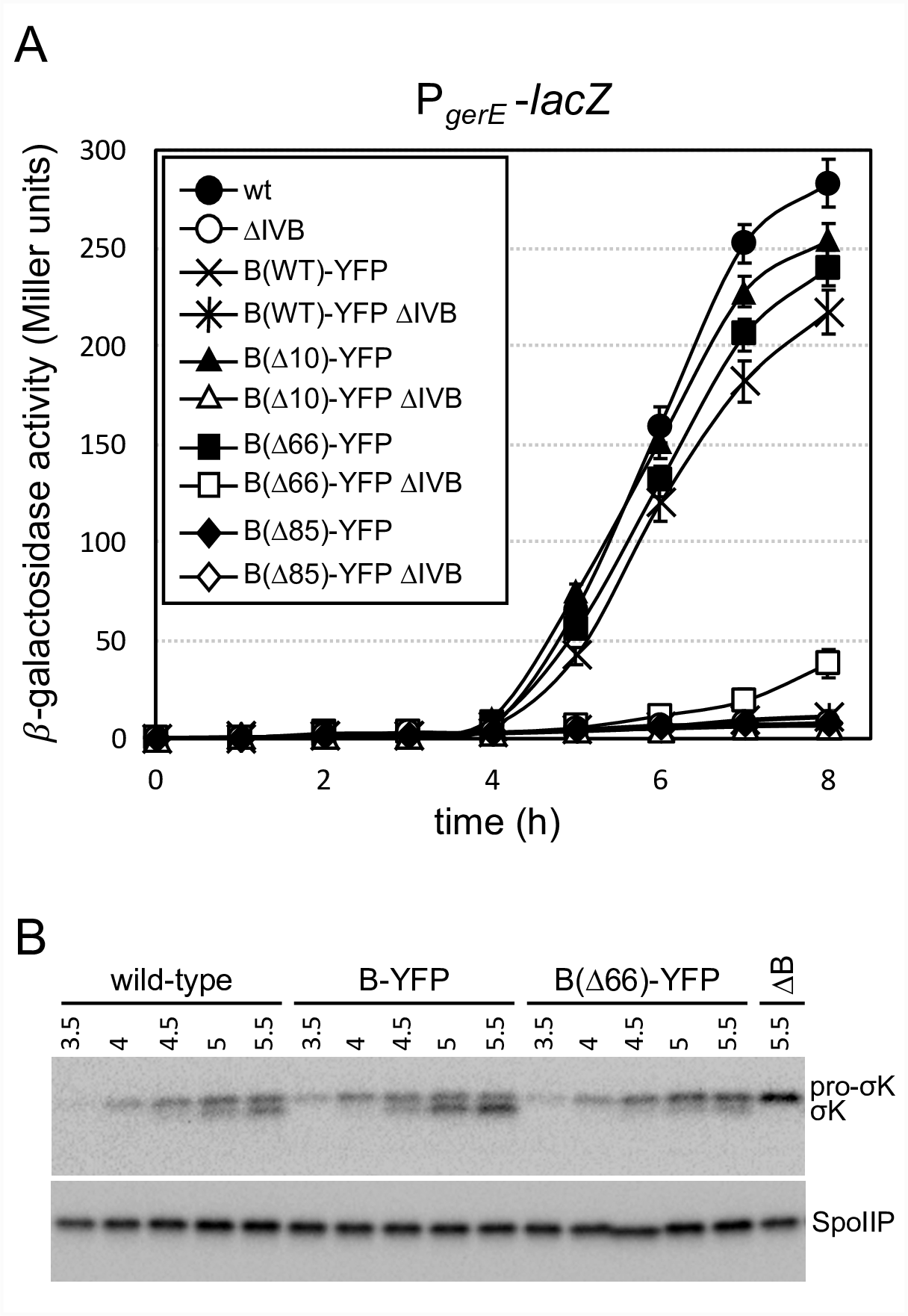

Supplement: S9 Fig — (A) σK activity was monitored using a σK-responsive promoter (PgerE) fused to lacZ. β-galactosidase activity was determined during a sporulation time course in the indicated strains (n = 3). Time (in hours) after the initiation of sporulation is indicated. In the presence of IVB, σK activity is induced with similar kinetics to wild-type in strains harboring wild-type B-YFP, B(Δ10)-YFP, and B(Δ66)-YFP but not B(Δ85)-YFP. In the absence of IVB, σK is not significantly activated. (B) Immunoblot of the indicated strains monitoring pro-σK processing during a sporulation time course. Time (in hours) after the initiation of sporulation is indicated above the blots. (top) B(Δ66)-YFP processes pro-σK to mature σK at a similar stage and to a similar extent as wild-type but slightly delayed compared to a match wild-type control (B-YFP). (bottom) SpoIIP is shown to control for loading. The initiation of sporulation was delayed by ~45 minutes in these experiments compared to the ones used for cytological analysis, however because these cultures were sporulated side-by-side the timing of σK activity and pro-σK processing can be compared. (TIF) [file pgen.1007753.s010.tif]

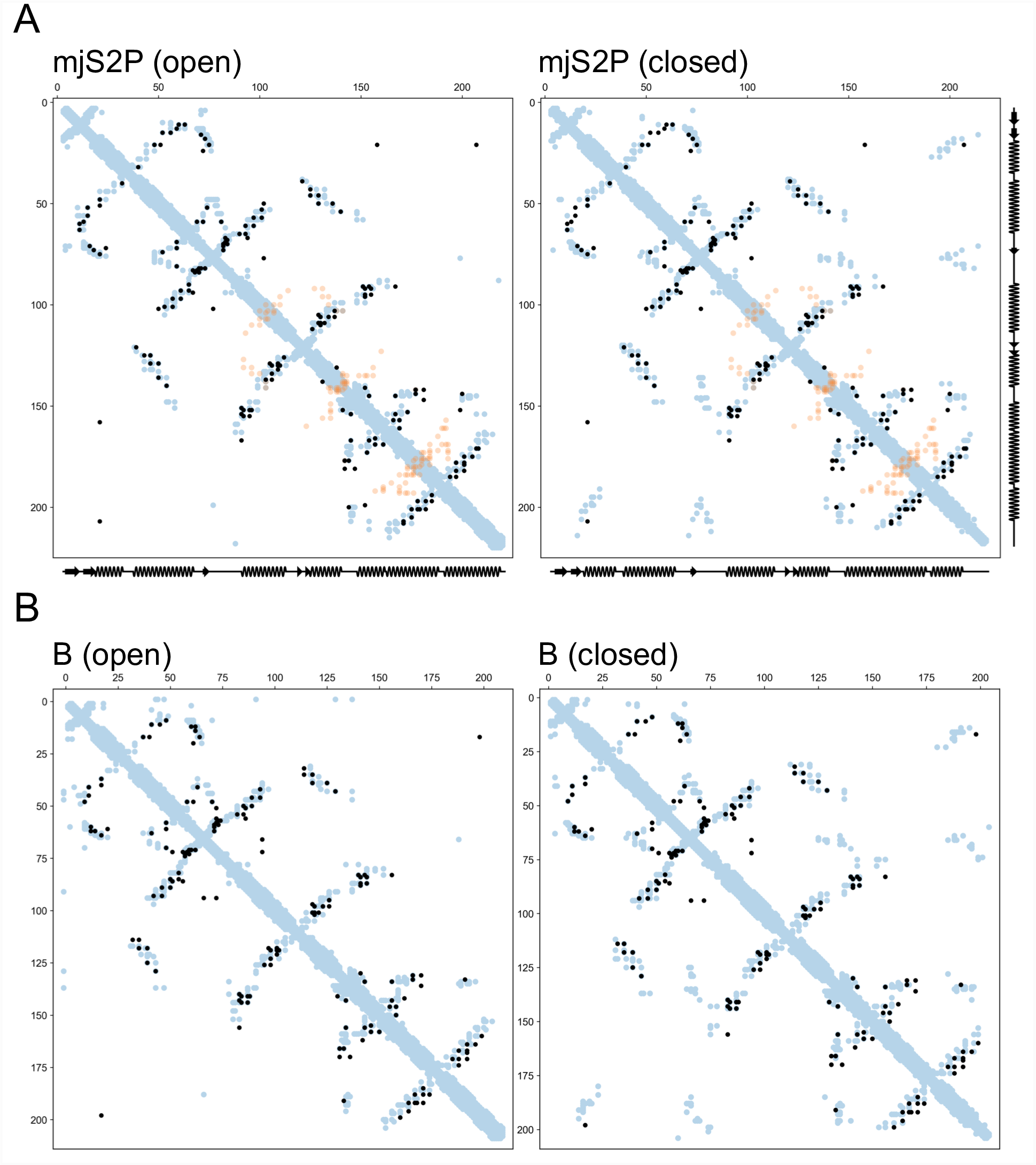

Supplement: S10 Fig — (A) Interaction maps showing evolutionary couplings (ECs) (black dots) compared to true contacts (blue dots) (<5 Å) based on the crystal structure of mjS2P (PDB 3B4R) in the open (chain A) and closed (chain B) conformations. Orange dots show interactions (<5 Å) between the two mjS2P molecules in the asymmetric unit of the crystal that form an anti-parallel pseudodimer. ECs shown (86 total) were determined at a probability threshold of 90%. (B) Co-variation analysis of the B protease. Interaction map showing 81 ECs (black dots) at a probability threshold of 90%. Blue dots indicate residue contacts within 5 Å of each other, determined from the homology models in the open and closed conformations. Axes represent sequence indices. (TIF) [file pgen.1007753.s011.tif]

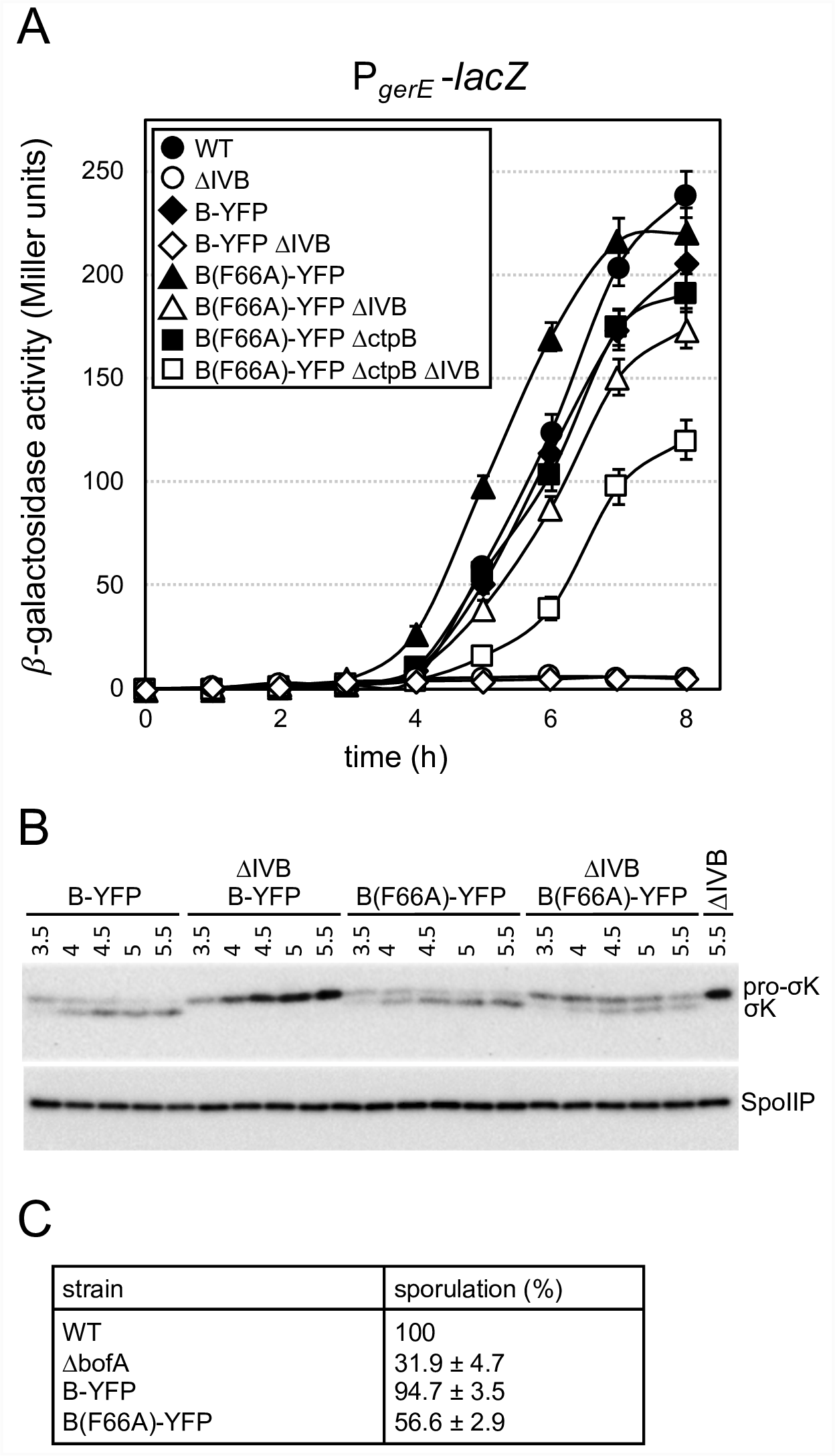

Supplement: S11 Fig — (A) σK activity was monitored using a σK-responsive promoter (PgerE) fused to lacZ. Liquid β-galactosidase assay during a sporulation time course in the indicated strains (n = 3). Only B(F66A)-YFP activates σK in the absence of IVB (open triangles) and in the absence of both IVB and CtpB (open squares). The initiation of sporulation was delayed by ~45 minutes in these experiments compared to the ones used for cytological analysis, however because these cultures were sporulated side-by-side the timing of σK activity can be compared. (B) Immunoblot analysis of pro-σK processing in the same strains used in (A). Time (in hours) after the initiation of sporulation is shown above the blot. SpoIIP immunoblot is shown to control for loading. (C) Sporulation efficiency is modestly reduced in cells lacking BofA or harboring B(F66A). Sporulation efficiencies of the indicated strains (n = 3) are shown. (TIF) [file pgen.1007753.s012.tif]

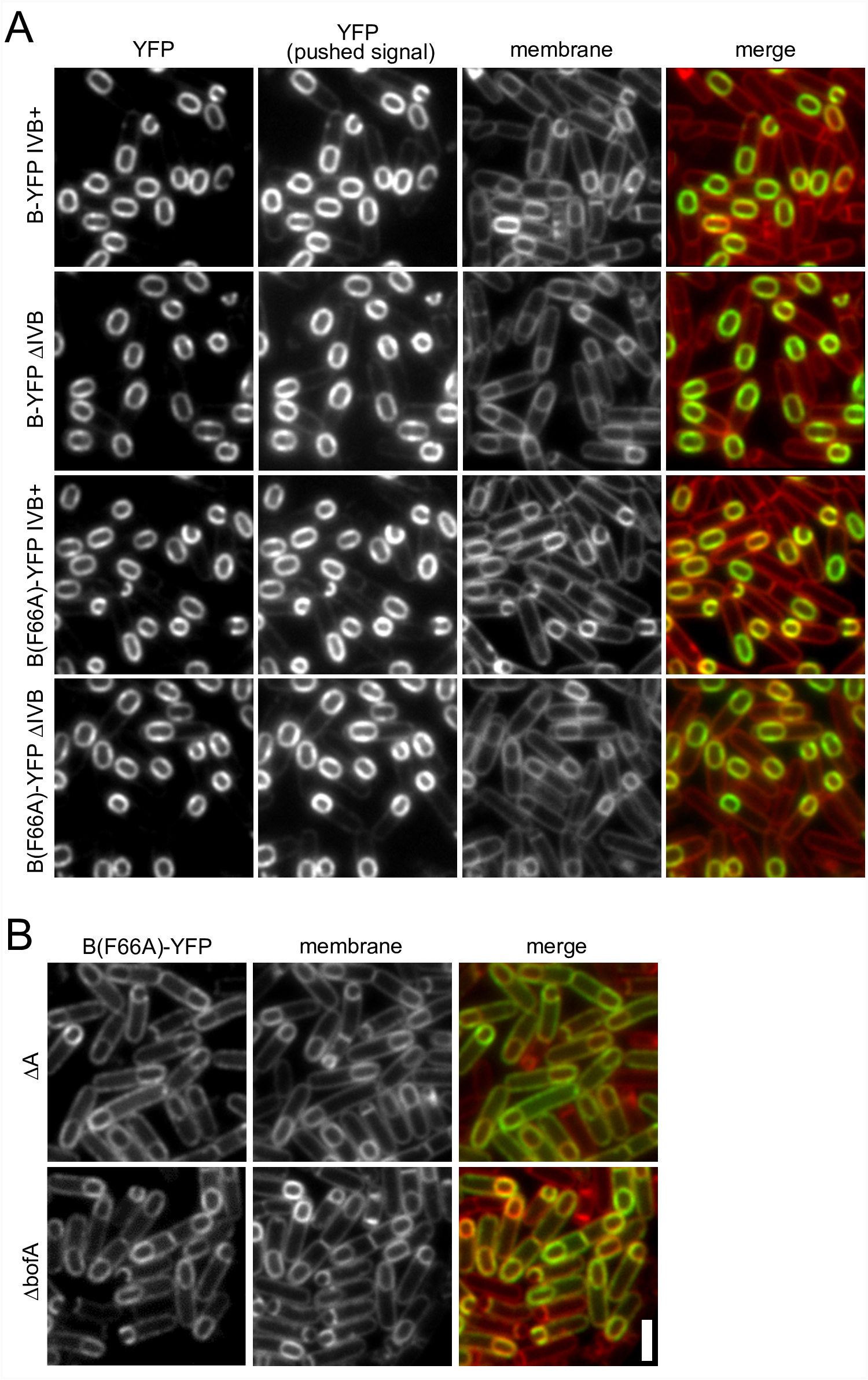

Supplement: S12 Fig — Representative images of the indicated strains at hour 4.5 of sporulation. B(F66A)-YFP localizes to the membranes surrounding the forespore in the presence and absence of the IVB protease (A) but not in sporulating cells lacking A or BofA (B). To more easily compare B-YFP signal in the mother-cell peripheral membranes the images were re-scaled to reveal weaker signals (pushed signal). Membranes were stained with the fluorescent dye TMA-DPH. Images from each strain were scaled identically. Scale bar indicate 2 μm. (TIF) [file pgen.1007753.s013.tif]

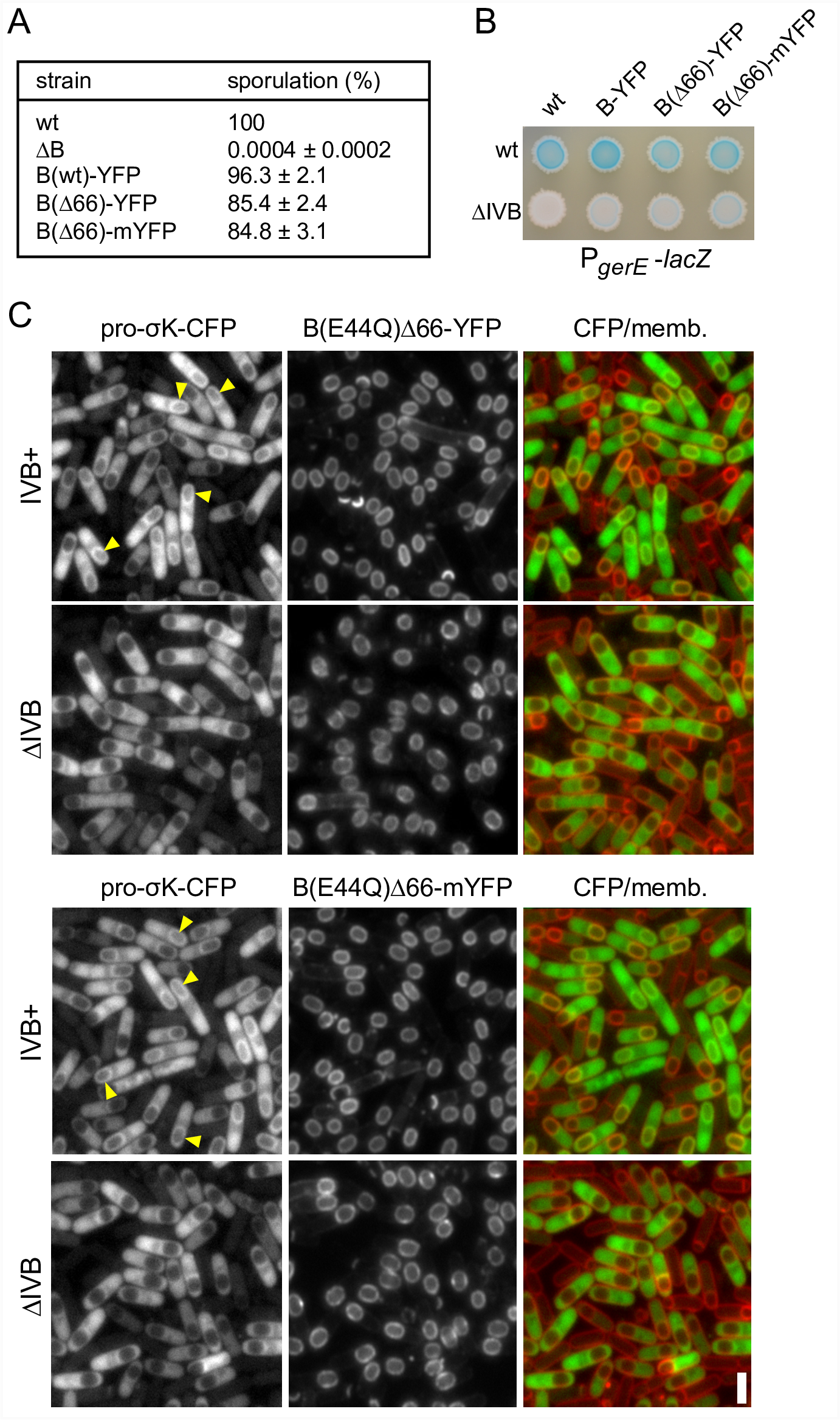

Supplement: S13 Fig — (A) Sporulation efficiency of the indicated strains (n = 3). (B) σK activity was monitored using a σK-responsive promoter (PgerE) fused to lacZ. Image of a sporulation agar plate containing X-gal with the indicated strains after 24 hours of incubation at 37˚C. (C) Representative images of the indicated strains at hour 4.5 of sporulation. Localization of pro-σK-CFP around the forespore (yellow carets) in strains with an intact IVB (site-1) signaling protease is indicated. Membranes were stained with the fluorescent dye TMA-DPH. Images were scaled identically. Scale bar indicates 2 μm. (TIF) [file pgen.1007753.s014.tif]
