# Supplementary material for: Evidence that regulation of intramembrane proteolysis is mediated by substrate gating during sporulation in Bacillus subtilis
Source: PLoS Genet. 2018 Nov 7;14(11):e1007753. doi: 10.1371/journal.pgen.1007753 (PMC6242693; doi:10.1371/journal.pgen.1007753)
Supplement: S1 Table — All strains, their genotypes, and sources are listed in this table. (PDF) [file pgen.1007753.s015.pdf]

S1 Table. *Bacillus subtilis* strains used in this study

Strain	Genotype	Source
PY79	Prototrophic wild-type	Youngman *et al.,* 1983
BNC279	*∆bofA::tet*	Campo & Rudner, 2006
BNC689	*∆spoIVB::phleo*	Campo & Rudner, 2006
BKM880	*spoIVFB-yfp (spec)*	This work
BKM846	*spoIVF::cat, amyE::spoIVFAB(E44Q)-gfp (spec) (tet)*	This work
BKM868	*spoIVF::cat, amyE::spoIVFAB(E44Q)-gfp (spec) (tet), spoIVB::erm*	This work
BKM1020	*spoIVF::cat, amyE::spoIVFAB(E44Q)-gfp (spec) (tet), spoIVC::erm*	This work
BKM1351	*ycgO::PspoIVF-spoIVFB (erm)*	This work
BKM1411	*spoIIIC-cfp (cat)*	This work
BKM1425	*spoIVF::cat::neo, ycgO::PspoIVF-spoIVFB(E44Q)-yfp (erm), spoIIIC-cfp(cat)*	This work
BKM1501	*spoIVF::cat::neo, ycgO::PspoIVF-spoIVFB(E44Q)-yfp (erm), spoIIIC-cfp(cat), amyE::spoIVFA (spec)*	This work
BKM1507	*spoIVF::cat::neo, ycgO::PspoIVF-spoIVFB(E44Q)-yfp (erm), spoIIIC-cfp(cat), amyE::spoIVFA (spec) spoIVB::phleo*	This work
BKM1557	*spoIVFAB::cat::neo, spoIIIC-cfp (cat)*	This work
BKM1595	*spoIVFAB::cat::neo, ycgO::PspoIVF-B(E44Q)-yfp (erm), spoIIIC-cfp (cat), amyE::spoIVFA (spec), bofA::tet*	This work
BCR1604	*spoIIIC-cfp (cat), spoIVFB-yfp (spec)*	This work
BCR1606	*spoIIIC-cfp (cat), spoIVFB-yfp (spec), spoIVB::phleo*	This work
BCR1621	*spoIVF::cat::neo, ycgO::PspoIVF-B(E44Q)-yfp (erm), spoIIIC-cfp (cat), bofA::tet*	This work
BCR1635	*amyE::Pspank-pro-sigK-cfp (spec)*	This work
BCR1639	*ycgO::Pspank-spoIVFB(E44Q)-yfp (erm), amyE::Pspank-pro-sigK-cfp (spec)*	This work
BCR1641	*ycgO::Pspank-spoIVFB-yfp (erm), amyE::Pspank-pro-sigK-cfp (spec)*	This work
BCR1646	*spoIIIC-cfp (cat), spoIVFB::spec*	This work
BCR1658	*spoIVFAB::cat::neo, ycgO::PspoIVF-spoIVFB(E44Q)∆10-yfp (erm), spoIIIC-cfp (cat), amyE::spoIVFA (spec)*	This work
BCR1659	*spoIVFAB::cat::neo, ycgO::PspoIVF-spoIVFB(E44Q)∆66-yfp (erm), spoIIIC-cfp (cat), amyE::spoIVFA (spec)*	This work
BCR1660	*spoIVFAB::cat::neo, ycgO::PspoIVF-spoIVFB(E44Q)∆85-yfp (erm), spoIIIC-cfp (cat), amyE::spoIVFA (spec)*	This work
BDR63	*spoIVF∆AB::cat*	Lu & Kroos, 1994
BDR64	*∆spoIVFB::spec*	Resnekov *et al.,* 1996
BDR622	*∆ctpB::tet*	Pan *et al.,* 2003
BDR1454	*spoIVB::erm, amyE::spoIVB(S378A) (cat)*	Campo & Rudner 2006
BDR3565	*∆spoIVFB::spec ycgO::cat*	This work
BDR3787	*∆spoIVFB::spec ycgO::PspoIVF-spoIVFB-yfp (erm)*	
BDR3568	*∆spoIVFB::spec ycgO::PspoIVF-spoIVFB∆10-yfp (erm)*	This work
BDR3569	*∆spoIVFB::spec ycgO::PspoIVF-spoIVFB∆66-yfp (erm)*	This work
BDR3570	*∆spoIVFB::spec ycgO::PspoIVF-spoIVFB∆85-yfp (erm)*	This work
BDR169	*spβ::gerE-lacZ (erm, cat)*	Losick collection
BDR3681	*spoIVFB-yfp (spec) spβ::gerE-lacZ(erm, cat)*	This work
BDR3682	*∆spoIVFB::spec ycgO::PspoIVFB-spoIVFB∆10-yf (erm) spβ::gerE-lacZ (erm, cat)*	This work
BDR3683	*∆spoIVFB::spec ycgO::PspoIVFB-spoIVFB∆66-yfp(erm) spβ::gerE-lacZ (erm, cat)*	This work
BDR3684	*∆spoIVFB::spec ycgO::PspoIVFB-spoIVFB∆85-yfp(erm) spβ::gerE-lacZ (erm, cat)*	This work
BDR3685	*∆spoIVB::kan*	This work
BDR3687	*spoIVFB-yfp (spec) ∆spoIVB::kan spβ::gerE-lacZ (erm, cat)*	This work
BDR3689	*∆spoIVFB::spec ycgO::PspoIVF-spoIVFB∆10-yfp (erm) spβ::gerE-lacZ (erm, cat) ∆spoIVB::kan*	This work
BDR3691	*∆spoIVFB::spec ycgO::PspoIVF-spoIVFB∆66-yfp (erm) spβ::gerE-lacZ (erm, cat) ∆spoIVB::kan*	This work
BDR3693	*∆spoIVFB::spec ycgO::PspoIVF-spoIVFB∆85-yfp (erm) spβ::gerE-lacZ (erm, cat) ∆spoIVB::kan*	This work
BDR3698	*spβ::gerE-lacZ (erm, cat) ∆spoIVB::kan*	This work
BDR3729	*ycgO::Pspank-spoIVF-yfp (erm), amyE::Pspank-pro-sigK-cfp (spec), yvbJ::Physpank-spoIVFA (cat), yhdG::Physpank-bofA (kan)*	This work
BDR3763	*lacA::spoIVB(S378A) (tet)*	This work
BDR3765	*spoIVFAB::cat::neo, ycgO::PspoIVF-spoIVFB(E44Q)-yfp (erm), spoIIIC-cfp (cat), amyE::spoIVFA (spec), spoIVB::phleo, lacA::spoIVB(S378A) (tet)*	This work
BDR3791	*∆spoIVFB::spec ycgO::PspoIVF-spoIVFB∆66-myfp (erm)*	This work
BDR3799	*spoIVFAB::cat::neo ycgO::PspoIVF-spoIVFB(E44Q)∆66-yfp (erm) spoIIIC-cfp (cat), amyE::spoIVFA (spec), spoIVB::phleo*	This work
BDR3802	*spoIVFAB::cat::neo ycgO::PspoIVF-spoIVFB(E44Q)∆66-myfp (erm), spoIIIC-cfp (cat), amyE::spoIVFA (spec)*	This work
BDR3804	*spoIVFAB::cat::neo, ycgO::PspoIVF-spoIVFB(E44Q)∆66-myfp (erm), spoIIIC-cfp (cat), amyE::spoIVFA (spec), spoIVB::phleo*	This work
BDR3810	*∆spoIVFB::spec ycgO::PspoIVF-spoIVFB∆66-myfp (erm) spβ::gerE-lacZ (erm, cat)*	This work
BDR3820	*∆spoIVFB::spec ycgO::PspoIVF-spoIVFB∆66-myfp (erm) spβ::gerE-lacZ (erm, cat) ∆spoIVB::kan*	This work
BDR3829	*∆spoIVFB::spec ycgO::PspoIVF-spoIVFB(F66A)-yfp (erm)*	This work
BDR3832	*∆spoIVFB::spec ycgO::PspoIVF-spoIVFB(F66A)-yfp (erm) ∆spoIVB::kan*	This work

BDR3836	*ΔspoIVFB::spec ycgO::PspoIVF-spoIVFB(F66A)-yfp (erm) spβ::gerE-lacZ (erm, cat)*	This work
BDR3839	*ΔspoIVFB::spec ycgO::PspoIVF-spoIVFB(F66A)-yfp (erm) spβ::gerE-lacZ (erm, cat) ΔspoIVB::kan*	This work
BDR3847	*ΔspoIVFB::spec ycgO::PspoIVF-spoIVFB(F66A)-yfp (erm) spβ::gerE-lacZ (erm, cat) ΔctpB::tet*	This work
BDR3849	*ΔspoIVFB::spec ycgO::PspoIVF-spoIVFB(F66A)-yfp (erm) spβ::gerE-lacZ (erm, cat) ΔctpB::tet ΔspoIVB::kan*	This work
BDR3856	*ΔctpB::tet spβ::gerE-lacZ (erm, cat)*	This work
BDR3857	*ΔctpB::tet ΔspoIVB::kan spβ::gerE-lacZ (erm, cat)*	This work
BDR3858	*spoIVFB-yfp ΔctpB::tet (spec) spβ::gerE-lacZ (erm, cat)*	This work
BDR3859	*spoIVFB-yfp ΔctpB::tet ΔspoIVB::kan (spec) spβ::gerE-lacZ (erm, cat)*	This work
BDR3864	*ΔspoIVFB::spec ycgO::PspoIVF-spoIVFB-yfp (erm) ΔspoIVB::kan*	This work
BDR3866	*spoIVFAB::cat::neo, ycgO::PspoIVF-spoIVFB(E44Q, F66A)-yfp (erm), spoIIIC-cfp (cat), amyE::spoIVFA (spec)*	This work
BDR3867	*spoIVFAB::cat::neo, ycgO::PspoIVF-spoIVFB(E44Q, F66A)-yfp (erm), spoIIIC-cfp (cat), amyE::spoIVFA (spec) spoIVB::phleo*	This work
BDR4044	*spoIIIC-cfp (cat), spoIVFB-yfp (spec), spoIVB::phleo, lacA::spoIVB(S378A) (tet)*	This work
BDR4047	*ΔspoIVFB::spec ycgO::PspoIVF-spoIVFB(Δ66) (erm)*	This work
BDR4051	*ΔspoIVFB::spec ycgO::PspoIVF-spoIVFB (erm)*	This work
