# Supplementary material for: Evidence that regulation of intramembrane proteolysis is mediated by substrate gating during sporulation in Bacillus subtilis
Source: PLoS Genet. 2018 Nov 7;14(11):e1007753. doi: 10.1371/journal.pgen.1007753 (PMC6242693; doi:10.1371/journal.pgen.1007753)
Supplement: S2 Table — All plasmids and their sources are listed in this table. (PDF) [file pgen.1007753.s016.pdf]

S2 Table. Plasmids used in this study

Plasmids	Description	Source
pKM190	*spoIVFB-yfp (spec)* (single crossover integration)	This work
pKM261	*ycgO::PspoIVF-spoIVFB(E44Q)-yfp (erm)*	This work
pKM266	*spoIIIC-cfp (cat)* (single crossover integration)	This work
pKM283	ycgO::PspoIVF-*spoIVFB-yfp (erm)*	This work
pCR275	*ycgO::Pspank-spoIVFB(E44Q)-yfp (erm)*	This work
pCR276	*ycgO::Pspank-spoIVFB-yfp (erm)*	This work
pCR278	*amyE::Pspank-pro-sigK-cfp (spec)*	This work
pCR286	*ycgO::PspoIVF-spoIVFB(E44Q)Δ10-yfp (erm)*	This work
pCR287	*ycgO::PspoIVF-spoIVFB (E44Q)Δ66-yfp (erm)*	This work
pCR288	*ycgO::PspoIVF-spoIVFB (E44Q)Δ85-yfp (erm)*	This work
pFR20	*ycgO::PspoIVF-spoIVFBΔ10-yfp (erm)*	This work
pFR21	*ycgO::PspoIVF-spoIVFBΔ66-yfp (erm)*	This work
pFR22	*ycgO::PspoIVF-spoIVFBΔ85-yfp (erm)*	This work
pFR28	*yvbJ::Phyperspank-spoIVFA (cat)*	This work
pFR29	*yhdG::Phyperspank-bofA (kan)*	This work
pFR30	*lacA::spoIVB(S378A) (tet)*	This work
pFR31	*ycgO::PspoIVF-spoIVFB(E44Q)Δ66-myfp (erm)*	This work
pFR32	*ycgO::PspoIVF-spoIVFBΔ66-myfp (erm)*	This work
pFR36	*ycgO::PspoIVF-spoIVFBΔ66 (erm)*	This work
pCB061	*ycgO::PspoIVF-spoIVFB(F66A)-yfp (erm)*	This work
