# Supplementary material for: Evidence that regulation of intramembrane proteolysis is mediated by substrate gating during sporulation in Bacillus subtilis
Source: PLoS Genet. 2018 Nov 7;14(11):e1007753. doi: 10.1371/journal.pgen.1007753 (PMC6242693; doi:10.1371/journal.pgen.1007753)
Supplement: S3 Table — All oligonucleotides used for plasmid construction, gene deletion, or sequencing are listed in this table. Capital letters were used for restriction endonuclease recognition sites and underlined letters indicate mutated bases. (PDF) [file pgen.1007753.s017.pdf]

S3 Table. Oligonucleotide primers used in this study

Primers	Sequence*
oDR078	gccGGATCCttatttgtatagttcatccatgcc
oDR079	gcgCTCGAGggttccggaatgag
oDR106	ggcAAGCTTacataaggaggaactactatgaataaatggctcgaccttatc
oDR481	gcggCAATTGcgtcgctctgcttctgcctgc
oDR482	cggCTCGAGgtagggcagaagcagttcctc
oDR594	gccGAATTCgggaatgaaataagcctgattgacg
oDR595	cggCTCGAGtttccccttcgccttcttccg
oCR599	gcgAAGCTTacataaggaggaactactatggtgacaggtgttttcgcagc
oCR600	cgcGCTAGCtttccccttcgccttcttccgctt
oCR601	cgcGCTAGCatggtttcaaaaggcgaagaactgt
oCR603	gcgAAGCTTacataaggaggaactactatgaataaatggctcgaccttat
oCR604	cgcGCTAGCttatttgtatagttcatccatgccatg
oCR605	cgcGCATGCttatttccccttcgccttcttccgct
oCR606	cgcGCATGCttacttataaagttcgtccatgccaag
oCR619	cggCTCGAGattcgtccgcttatcggcaaag
oCR620	cggCTCGAGctccctgtttttccgtaatacc
oCR621	cggCTCGAGgtgcctttgccgatattcctc
oFR48	gaagggtaccagcggtatgt
oFR49	ctgagcgagggagcagaaatcgggcattcactact
oFR50	gttgaccagtgctccctggcaagctgactgccgga
oFR51	gaatggaccaagagtgcagg
oFR58	gagcggataacaattaagcttacataaggaggaactactatgagtcacagagcagatga
oFR59	ttagcttgcatgcgagctagcttattcaaatgaaatcacc
oFR62	cggACTAGTacataaggaggaactactatggagcctatttttattattggg
oFR66	gccGCATGCttaaatgataaattgcttaatgacgactaa
oFR72	gccCTCGAGttgccgatgataagctgtc
oFR73	gccGCTAGCActggtaatggtagcgaccg
oFR77	gccCTCGAGggttccggagtttcaaaaggcgaagaactg
oFR78	gccGGATCCttacttataaagttcgtccatgcc
oFR83	ggcGGATCCTTActcaagctccctgtttttccgta
oFR84	ggcAAGCTTacataaggaggaactactatgaataaatggctcgaccttatc
oCB038	gaatcaagcgtgtttttttgctgccggctggcggaacggtcgaagtggaag
oCB039	cttccacttcgaccgttccgccagccggcagcaaaaaaacacgcttgattc

* Capital letters indicate restriction endonuclease sites and underlined letters indicate mutated bases
